# Highly Sensitive Measurement of Horseradish Peroxidase Using Surface-Enhanced Raman Scattering of 2,3-Diaminophenazine

**DOI:** 10.3390/molecules29040793

**Published:** 2024-02-08

**Authors:** Evgeniy G. Evtushenko, Elizaveta S. Gavrilina, Alexandra D. Vasilyeva, Lyubov V. Yurina, Ilya N. Kurochkin

**Affiliations:** 1 N.M. Emanuel Institute of Biochemical Physics RAS, Kosygina Str. 4, 119334 Moscow, Russiaalexandra.d.vasilyeva@gmail.com (A.D.V.); ikur@genebee.msu.ru (I.N.K.); 2 Faculty of Chemistry, Lomonosov Moscow State University, Leninskie Gory 1/3, 119991 Moscow, Russia

**Keywords:** surface-enhanced Raman scattering, SERS, horseradish peroxidase, HRP, ELISA, 2,3-diaminophenazine, DAP, *o*-phenylenediamine, 1,2-diaminobenzene, oPD

## Abstract

The development of various enzyme-linked immunosorbent assays (ELISAs) coupled with surface-enhanced Raman scattering (SERS) detection is a growing area in analytical chemistry due to their potentially high sensitivity. A SERS-based ELISA with horseradish peroxidase (HRP) as an enzymatic label, an *o*-phenylenediamine (oPD) substrate, and a 2,3-diaminophenazine (DAP) enzymatic product was one of the first examples of such a system. However, the full capabilities of this long-known approach have yet to be revealed. The current study addresses a previously unrecognized problem of SERS detection stage performance. Using silver nanoparticles and model mixtures of oPD and DAP, the effects of the pH, the concentration of the aggregating agent, and the particle surface chloride stabilizer were extensively evaluated. At the optimal mildly acidic pH of 3, a 0.93 to 1 M citrate buffer, and AgNPs stabilized with 20 mM chloride, a two orders of magnitude advantage in the limits of detection (LODs) for SERS compared to colorimetry was demonstrated for both DAP and HRP. The resulting LOD for HRP of 0.067 pmol/L (1.3 amol per assay) underscores that the developed approach is a highly sensitive technique. We suppose that this improved detection system could become a useful tool for the development of SERS-based ELISA protocols.

## 1. Introduction

An enzyme-linked immunosorbent assay (ELISA) with colorimetric detection is a universal analytical platform used for the quantitative measurement of various antigens. Although highly successful and widely used, in some cases, it suffers from a limited sensitivity, as more and more antigens emerge (e.g., clinically relevant ones) that have to be detected at very low concentrations. One of the natural solutions for such instances is the replacement of colorimetry with more sensitive detection techniques such as fluorescence [1,2] or enhanced chemiluminescence [3]. Surface-enhanced Raman scattering (SERS) is also a promising candidate that meets the requirement of high sensitivity. Indeed, a SERS-based ELISA is a growing area in analytical chemistry that is gradually accumulating a range of available approaches as well as a number of successful applications [4,5,6,7,8,9,10,11,12,13,14,15,16,17,18,19,20] (see also reviews [21,22]).

Horseradish peroxidase (HRP) is the most common enzyme label used in an ELISA. It is readily available both as a pure enzyme and conjugated with antibodies. Therefore, the HRP label is the first choice for the development of a more sensitive detection technique. A direct approach for the SERS-based HRP measurement relies on the difference in spectra for enzymatic substrate and product. For highly sensitive applications, the product should have a much more intense SERS spectrum compared to the substrate, as the latter is typically present in excess. In other words, the product should have both a high Raman scattering cross-section and a high affinity for the metal surface used for enhancement. Several substrates have been tested up to date: *o*-phenylenediamine (oPD) [14,15,23,24,25,26,27,28], 2,2′-azino-bis(3-ethylbenzothiazoline-6-sulfonic acid) (ABTS) [16], 3,3′,5,5′-tetramethylbenzidine (TMB) [17,18,19], and leuco dyes [11]. Among these, oPD appears to be the most promising. Firstly, it results in a well-defined and sufficiently soluble product: 2,3-diaminophenazine (DAP) [29,30]. Secondly, this reaction is not a simple oxidation, but rather, an oxidative dimerization, leading to substantial differences in the chemical structures, and hence, in the vibrational spectra of the substrate and the product. It has also been previously shown that all forms of DAP (neutral DAP, protonated DAPH^+^, and doubly-protonated DAPH_2_^2+^) exhibit intense SERS spectra with silver nanoparticles [15,27,31,32]. Finally, both oPD and DAP are commercially available, which is not relevant for the assay but greatly simplifies the research, as model solutions of both substances and their mixtures may be easily prepared and studied.

In general, the SERS-based measurements of HRP consist of two steps: enzymatic reaction and SERS detection of the resultant product. In order to achieve a high sensitivity, the conditions for both steps should be optimized. While several examples of enzymatic reaction optimization have been described previously for colorimetric- [33,34,35] and SERS-based measurements [15] of HRP with the oPD substrate, no studies have focused on understanding and refining the SERS detection procedure. For the first time, the current paper addresses this issue. Using silver nanoparticles (AgNPs) and model mixtures of oPD and DAP, we successively and rationally evaluated the effects of the pH, the concentration of the aggregating agent, and the particle surface chloride stabilizer. As a result, we strongly narrowed the range of optimal detection conditions to a mildly acidic pH of 3–4 using a 0.93–1 M citrate buffer and AgNPs stabilized with 20 mM chloride. With these optimal conditions, we have demonstrated a two orders of magnitude advantage in the detection limits of SERS compared to colorimetry for both DAP and HRP. The paper is intentionally rich in appendices, with some of them providing additional information about the behavior of the studied system and the remainder being dedicated to the detailed description of the experiments for easier reproduction of the protocol.

## 2. Results

### 2.1. Synthesis, Characterization, and Standardization of Silver Nanoparticles

**Figure 1:**
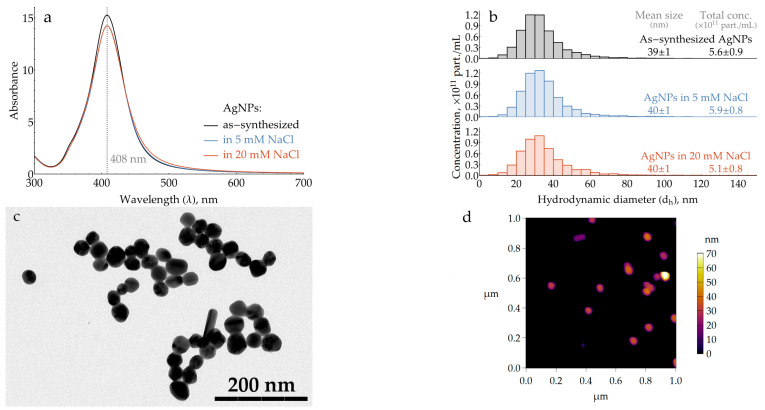
Characterization of AgNP colloids. (**a**) Typical UV–visible absorption spectra. (**b**) Hydrodynamic diameter and particle concentration measured with nanoparticle tracking analysis. Data for panels (**a**) and (**b**) are provided for both “as-synthesized” particles and those transferred into 5 or 20 mM NaCl. Numerical data in panel (**b**) are presented as 95% confidence intervals. (**c**) Transmission electron micrograph of AgNPs. (**d**) Atomic force topography of AgNPs on silanized silicon wafer.

Silver nanoparticles are known to be slowly oxidized by atmospheric oxygen. In order to obtain repeatable results over the entire study, fresh batches of AgNP colloids were routinely synthesized using a well-established hydroxylamine method [36]. To minimize oxidation, they were stored in a reaction medium containing excess hydroxylamine and used within 36 h after the synthesis. UV–vis spectra were used for the rapid characterization of each batch. To standardize the particle size, samples with a maximum of plasmonic band between 406.6 and 408.6 nm (Figure 1a) were selected for further use. The number-weighted mean hydrodynamic diameter of the synthesized particles, measured using nanoparticle tracking analysis (NTA), was found to be 39 ± 1 nm (Figure 1b). The particle shape was characterized using the combination of two techniques: transmission electron microscopy (TEM) and atomic force microscopy (AFM), as they complement each other. While TEM provides adequate information about the shape of a particle’s projection along the XY plane, it lacks data on the Z direction. On the other hand, although the extraction of a particle’s lateral dimensions from AFM data is not straightforward due to convolution with the tip shape and minor XY drifts of the sample, the heights of rigid silver particles on a rigid silicon support are measured with sub-nm accuracy. For the TEM data (Figure 1c), each particle’s projection (N = 500) was measured in two directions: the longest axis, with a mean of 39.1 nm and standard deviation (SD) of 7.8 nm, and the shortest one, with a mean of 33.9 nm and SD of 6.1 nm. The average aspect ratio was 1.16. The average particle height determined from AFM (Figure 1d) was 35.1 nm with an SD of 8.7 nm (N = 122). For additional information about the particle shape, please refer to Appendix A.

As long as the reaction medium contains excess hydroxylamine, which is a reactive compound that could cause some side reactions, it is preferred to transfer AgNPs into a standard medium for the SERS measurements. During the synthesis, particles are stabilized with a chloride anion [36] provided by hydroxylamine hydrochloride, so the most natural way is to keep this stabilizer and remove all other components. This was performed using double centrifugation and pellet resuspension in a NaCl solution. In order to standardize the particle concentration, the AgNP colloid absorption at the plasmonic maximum was adjusted to 14.25 (0.95 at a 15-fold dilution). As evidenced by the NTA data (Figure 1b), this procedure causes only a minor shift in the mean particle size from 39 to 40 nm, which is expected due to the loss of the smallest particles to the supernatant. The total particle concentration also does not change within the NTA technique uncertainty. The samples of standardized AgNPs were used for the SERS measurements within 5 h to prevent particle oxidation due to atmospheric oxygen.

### 2.2. Estimation of HRP Effective K_M_ for oPD

**Figure 2:**
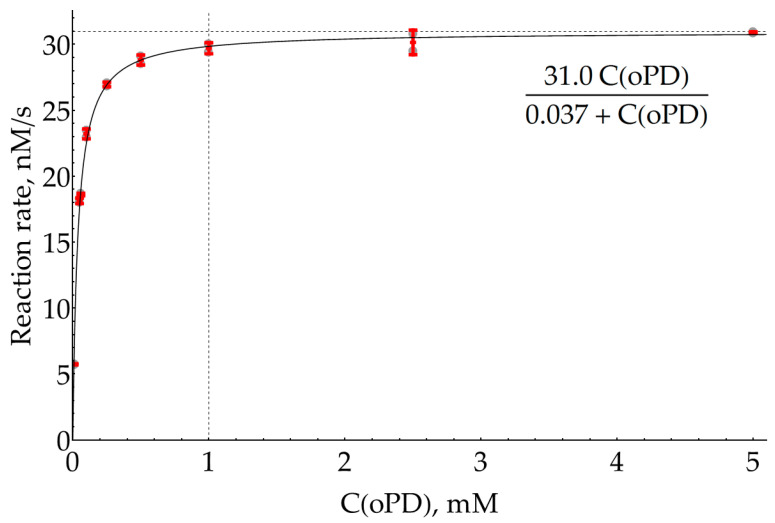
Dependence of HRP enzymatic reaction rate on oPD concentration measured colorimetrically at 421 nm in 100 mM sodium phosphate buffer (pH 6.0) with 8 ng/mL HRP and 80 μM H_2_O_2_. Open gray circles represent raw data (N = 2); red bars denote the mean ± standard deviation. A solid line represents the best fit.

Although we have claimed that our main goal is the optimization of SERS measurements of DAP, this procedure is dependent on the conditions of the preceding enzymatic reaction. SERS spectra with AgNPs were previously reported for both oPD [15,27,37,38] and DAP [15,27,31,32], implying that they both have some affinity for the silver surface, so during the SERS measurements, they compete for binding to the metal. Thus, our goal could be refined as a search for conditions that favor the binding of small quantities of enzymatically formed DAP over the excess of unreacted oPD. In order to estimate the highest concentration of oPD that could be passed to the SERS detection stage from the enzymatic stage, the effective Michaelis constant for oPD was measured colorimetrically at 421 nm in 100 mM sodium phosphate buffer (pH 6.0) and a H_2_O_2_ concentration of 80 μM, which has been previously reported to be optimal in terms of the background reaction rate [35]. The 95% confidence interval for K_M_ was found to be 37 ± 3 μM. From this value and also visually from the graph (Figure 2 and Appendix B), it could be concluded that an oPD concentration of 1 mM (around 27 × K_M_) is the reasonable maximum. Indeed, additional oPD causes an increase of only a few percent in the enzymatic product formation rate but interferes with DAP during a subsequent SERS detection step.

### 2.3. Selection of pH for SERS Detection of DAP

An HRP enzymatic reaction is usually performed at a pH of 5 to 7; however, sensitive SERS detection of the produced DAP in its mixtures with unreacted oPD may have a different optimal pH than from within this range. As long as DAP has a pronounced SERS spectrum over a broad range of pHs (see Appendix C), hypothetically, several strategies may be considered:

- The enzymatic reaction is stopped by an acid or base, and this mixture is further added to the AgNP colloid for SERS measurements. The stop reagent should create the optimal pH for DAP binding to the silver surface and the optimal ionic strength for the proper aggregation of AgNPs.
- If the sensitive detection of DAP in its mixtures with oPD is possible at the pH optimum of HRP, the enzymatic reaction mixture could be directly added to the AgNP colloid, followed by SERS detection. The advantage of this approach is the absence of dilution by the stop reagent.

In this section and related appendices, we extensively analyze which processes interfere with DAP detection at various pHs for the rational exclusion of unviable strategies.

The first factor is the presence of unreacted H_2_O_2_ in reaction mixtures, which could cause the partial dissolution of AgNPs [4,39,40]. Using spectrophotometric measurements of the position and absorbance in the plasmonic band maximum, it has been shown (see Appendix D) that at an alkaline pH of 9, a measurable dissolution of AgNPs occurs within 10 min of incubation with 100 μM H_2_O_2_. Thus, stopping the enzymatic reaction by shifting to an alkaline pH is an unfavorable strategy.

**Figure 3:**
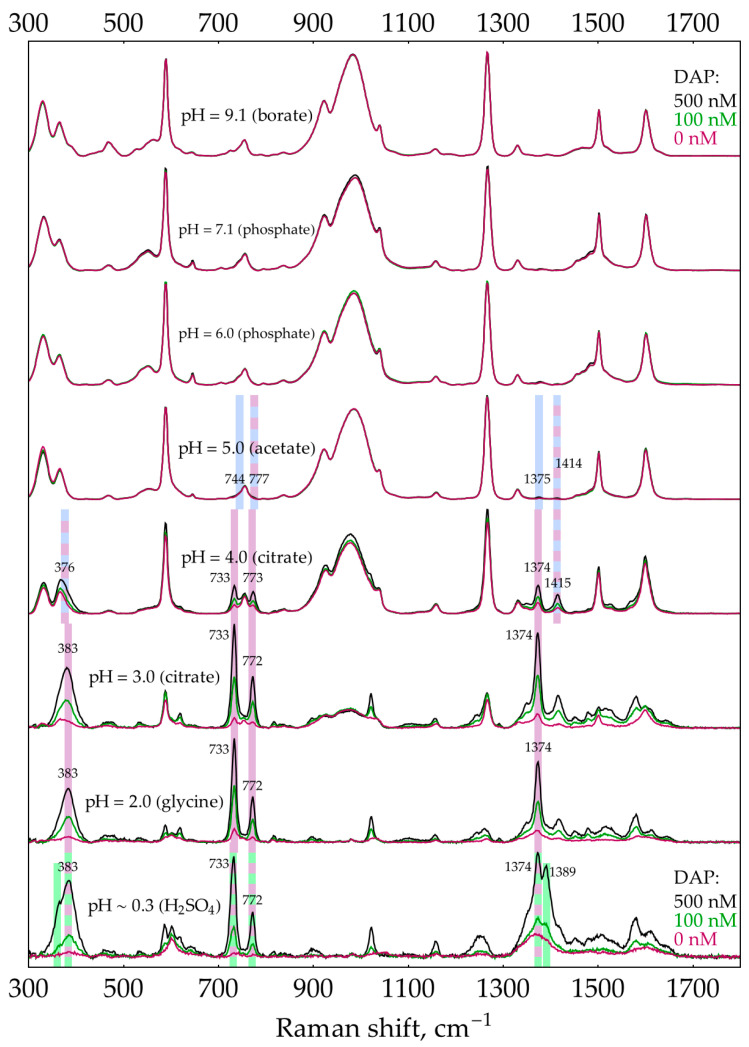
SERS spectra of 0, 100, and 500 nM DAP in the presence of 1 mM oPD with AgNPs in 5 mM NaCl at different pHs. The polynomial baseline was subtracted from each spectrum. All three spectra at each pH were normalized to the most intense band of all DAP concentrations at this pH. The shading and numbers represent the most intense bands of different forms of DAP: blue—DAP, purple—DAPH^+^, and green—DAPH_2_^2+^. For the reference spectra of oPD and DAP at different pHs, please refer to Figure A8 and Figure A15, respectively.

**Figure 4:**
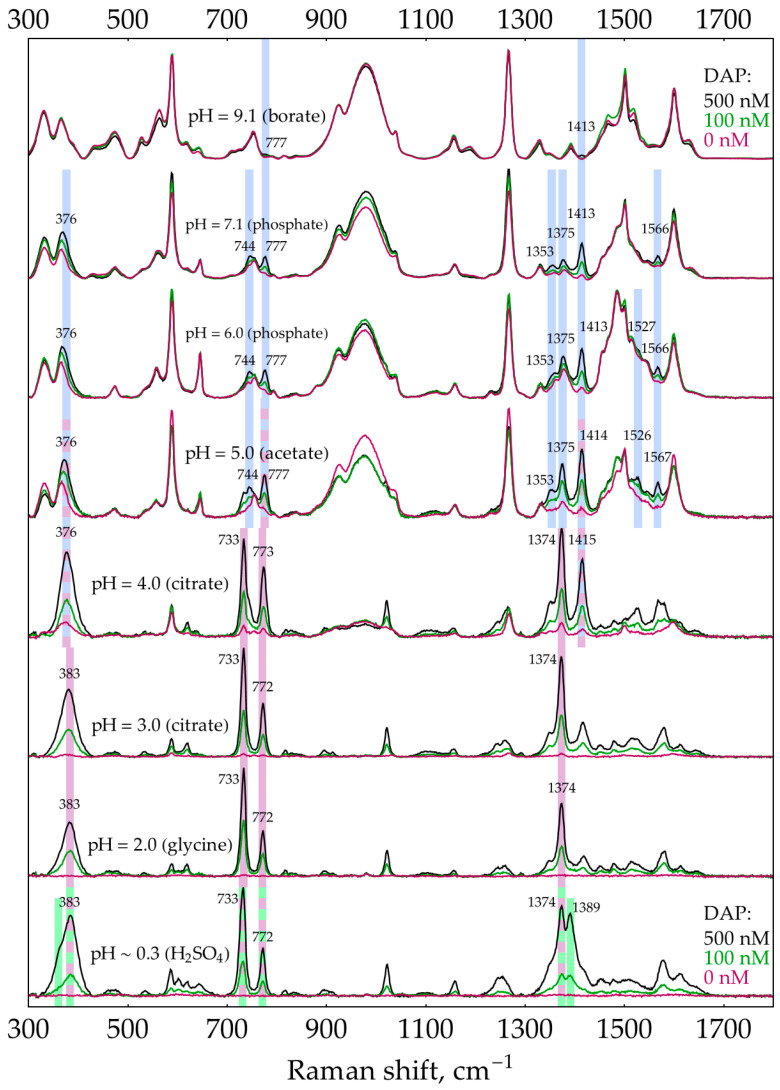
SERS spectra of 0, 100, and 500 nM DAP in the presence of 0.1 mM oPD with AgNPs in 5 mM NaCl at different pHs. The polynomial baseline was subtracted from each spectrum. All three spectra at each pH were normalized to the most intense band of all DAP concentrations at this pH. The shading and numbers represent the most intense bands of different forms of DAP: blue—DAP, purple—DAPH^+^, and green—DAPH_2_^2+^. For the reference spectra of oPD and DAP at different pHs, please refer to Figure A8 and Figure A16, respectively.

The second factor is the presence of unreacted oPD. A two-step process was used to estimate the feasibility of DAP detection in its mixtures with oPD at different pHs. During the first step, aimed at narrowing the range of prospective pHs, a screening was performed using default concentrations of buffers/media (0.32 M for pH 9.1 and 0.5 M for other pHs). The pH is considered prospective if it meets two criteria. First, the most intense DAP bands should be detectable at a moderate DAP concentration of 100 nM. Second, their intensity should increase at 500 nM DAP. The screening results (Figure 3) show that in the presence of 1 mM oPD, DAP could be detected at pHs from 4 down to a strongly acidic conditions. Notably, the sensitivity at this range is high enough to detect weak DAP bands in the absence of added DAP. They originate from the minor quantities of DAP present in stock oPD. At a higher pH, oPD dominates the SERS spectrum, with no detectable DAP bands. As long as 1 mM of oPD was determined to be the highest reasonable concentration to use in the enzymatic reaction, the same screening was also performed at a lower oPD concentration of 0.1 mM (Figure 4). The qualitative picture is the same, with the prospective range extended up to pH 7.1 due to the weaker competition of oPD with DAP for binding to the silver surface. Another important feature is that at pH ≥ 5, the oPD spectrum is concentration dependent and contains bands with poorly reproducible intensities, thus providing an unsteady background for DAP detection. All the features mentioned in the oPD spectra are discussed in detail in Appendix E.

**Figure 5:**
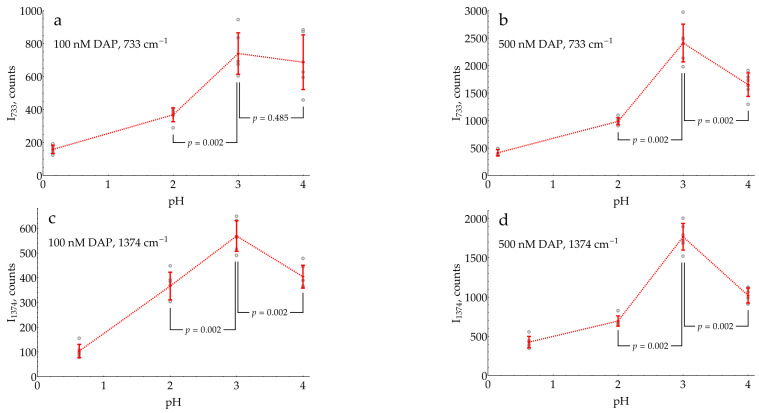
Comparison of SERS intensities of DAP bands in DAP mixtures with 1 mM oPD at different pHs. The concentrations of buffers/media used for AgNP aggregation are listed in Appendix F. For each data point, a signal from a corresponding blank sample without added DAP was subtracted. (**a**,**b**) SERS intensities at 733 cm^−1^ with 100 or 500 nM DAP, correspondingly; (**c**,**d**) SERS intensities at 1374 cm^−1^ with 100 or 500 nM DAP, correspondingly. Open gray circles represent raw data (N = 6); red bars denote mean ± standard deviation. Reported *p*-values result from the Mann–Whitney test. Dashed lines connect the means for visual clarity.

At the second step of pH selection, potential candidates from the screening (pH from 0.3 to 4) were directly compared to each other. In order to use the best possible conditions for each pH, the concentrations of buffers/media serving as aggregating agents for the AgNPs were optimized (Appendix F). The results of this comparison are shown in Figure 5.

The highest SERS signal for both of the most intense DAPH^+^ bands (733 and 1374 cm^−1^) in the presence of 1 mM oPD was observed at a mildly acidic pH of 3. Hence, the general procedure for the SERS detection of HRP activity should include the enzymatic reaction, stopped by the addition of concentrated citrate buffer with pH = 3 up to the final concentration of 0.93 M for the optimal aggregation of AgNPs. This sample could be directly mixed with AgNPs in a 1:1 ratio, with SERS measurement 2 min later.

### 2.4. Influence of AgNP Chloride Stabilizer

The last factor to consider in the current paper is the impact of AgNP surface modification on the SERS detection of DAP. This topic is truly inexhaustible, so only one system was studied as an example of this approach. The chloride anion has a strong affinity for the silver surface and provides the negative charge of AgNPs. In all previous experiments, AgNPs stabilized with 5 mM NaCl were used. This concentration could be varied in a narrow range from 2.5 to 20 mM, with AgNPs being unstable outside this range on a timescale of several hours. Below 2.5 mM, there is not enough chloride on the surface to fully stabilize the colloid. Above 20 mM, destabilization occurs due to the contraction of the electrical double layer by the increased ionic strength.

**Figure 6:**
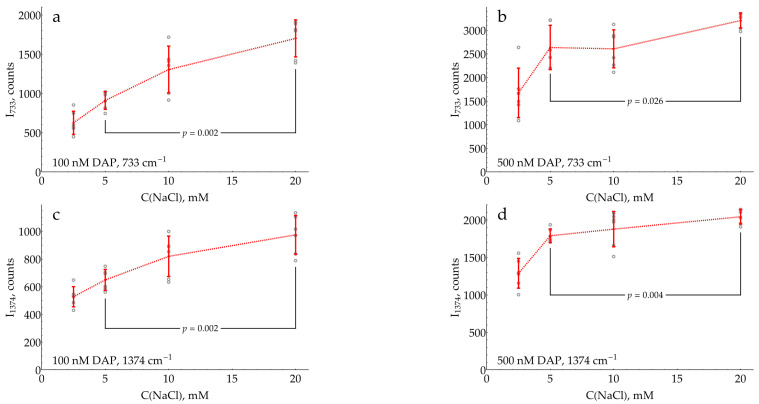
SERS intensities of DAP bands in DAP mixtures with 1 mM oPD, measured with AgNPs stabilized by varying NaCl concentrations and aggregated with 0.93 M of citrate buffer at pH 3. For each data point, a signal from a corresponding blank sample without added DAP was subtracted. (**a**,**b**) SERS intensities at 733 cm^−1^ with 100 or 500 nM DAP, respectively; (**c**,**d**) SERS intensities at 1374 cm^−1^ with 100 or 500 nM DAP, respectively. Open gray circles represent raw data (N = 6); red bars denote mean ± standard deviation. Reported *p*-values result from the Mann–Whitney test. Dashed lines connect the means for visual clarity.

The data provided in Figure 6 evidence that the SERS intensity for both DAP bands asymptotically increases with the saturation of the silver surface by chloride. The highest NaCl concentration of 20 mM, which still results in stable AgNPs, should be used in the assay.

### 2.5. Comparison of Colorimetric and SERS Detection Methods for DAP and HRP

**Figure 7:**
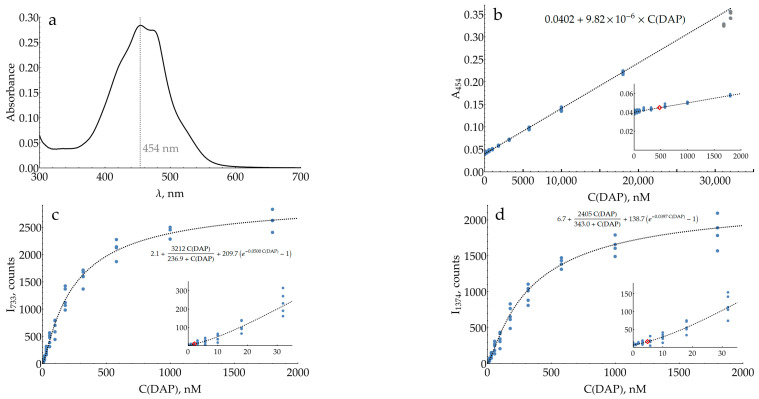
Measured absorption and SERS intensities for different DAP concentrations in DAP mixtures with 0.33 mM oPD in 0.93 M citrate buffer at pH 3. (**a**) Absorption spectrum of 18 μM DAP; (**b**) absorption at 454 nm measured using a plate reader with sample volumes of 200 μL (N = 29 for blanks and 8 for other concentrations); (**c**) SERS intensity at 733 cm^−1^ (N = 20 for blanks and 5 for other concentrations); (**d**) SERS intensity at 1374 cm^−1^ (N = 20 for blanks and 5 for other concentrations). Insets represent the regions of low concentrations, with the LOD marked with a red diamond. Dashed lines represent best fits.

In order to evaluate the performance of the optimized conditions for DAP measurement, colorimetric and SERS detection methods were directly compared to each other (Figure 7). Both assays were performed in a 0.93 M citrate buffer at pH 3. The oPD concentration was chosen to be 0.33 mM with the following considerations: The maximum concentration of the stock citrate buffer is around 1.5 M. At higher concentrations, it starts to precipitate if the room temperature drops below 20 °C. This obstacle strongly deteriorates the repeatability if thin and poorly visible white sediment is overlooked. Thus, to stop the enzymatic reaction, the sample should be diluted around three times with stock 1.5 M citrate, which turns 1 mM of oPD from the enzymatic reaction into 0.33 mM on a SERS detection stage. It should be noted that a pH of 3 is optimal not only for SERS but also for colorimetry, as the molar absorption coefficient of DAPH^+^ is slightly higher than that of DAP (21,400 vs. 17,000 M^−1^ × cm^−1^ [41]). Thus, the comparison of both techniques in these conditions is justified.

The calibration curve for colorimetric detection (Figure 7b) was linear, with a negative deviation at high DAP concentrations above 18 μM. The limit of detection (LOD) was estimated to be 478 nM. The mean coefficient of variation (CV) for the range above the LOD was 6%.

Both concentration curves for the SERS measurements of DAP (Figure 7c,d) were strongly nonlinear (S-shaped). Saturation at high concentrations is typical for SERS measurements due to the occupation of all available binding sites on the silver surface. A negative deviation from linearity at low concentrations is less expected. Two hypotheses could be proposed for this phenomenon. First, the issue might be kinetic: at low concentrations, two minutes is not enough to reach equilibrium for DAPH^+^ adsorption onto the silver surface. On the other hand, it could be an indication of a cooperative process, namely, the oligomerization of DAP on the surface, which has been shown for DAP on MoSe_2_ [42].

In order to describe these datasets with calibration curves, an appropriate function was found. It includes the exponential term to account for the concave shape at low concentrations, together with the hyperbolic term, characteristic of processes with saturation. The estimated LODs for the SERS detection of DAP were 2.2 and 4.9 nM for 733 and 1374 cm^−1^ bands, respectively. These values are around two orders of magnitude lower than the LOD of the colorimetric technique. The mean CVs for the range above the LOD were measured to be 24% and 22%, respectively.

**Figure 8:**
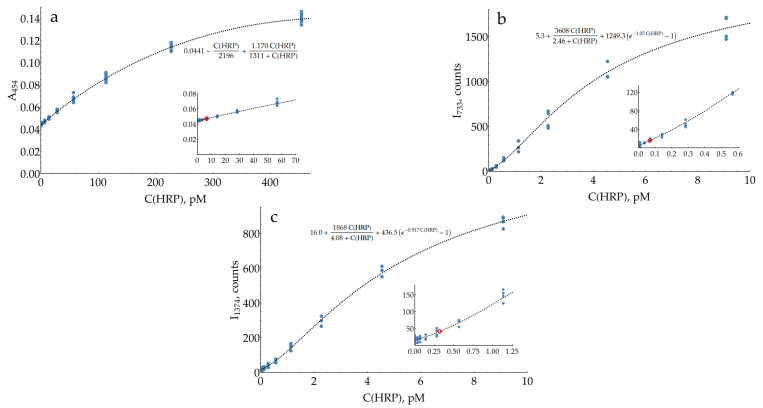
Absorbance and SERS intensities for different HRP concentrations. The enzymatic reaction was performed for 10 min at pH 6, 1 mM oPD, and 80 μM H_2_O_2_. It was stopped using three-fold dilution with 1.5 M citrate buffer at pH 3. (**a**) Colorimetric detection at 454 nm measured using a plate reader with total sample volumes of 200 μL (N = 12 for blanks and 8 for other concentrations); (**b**) SERS intensity at 733 cm^−1^ (N = 19 for blanks and 4 for other concentrations); (**c**) SERS intensity at 1374 cm^−1^ (N = 19 for blanks and 4 for other concentrations). Insets represent the regions of low concentrations, with the LOD marked with a red diamond. Dashed lines represent best fits.

Finally, the same comparison was made for HRP (Figure 8). As long as the optimization of the enzymatic reaction stage is beyond the scope of the current study, for this demonstration, some typical conditions were used: room temperature, pH 6, 1 mM oPD, 80 μM H_2_O_2_, and a reaction time of 10 min. The reaction was stopped using a three-fold dilution with citrate buffer (1.5 M at pH = 3). The chosen conditions influence both detection techniques (SERS and colorimetry) equally; hence, the estimate for the ratio of the two LODs is unbiased.

The calibration curve for the colorimetric HRP measurement (Figure 8a) was nonlinear, as reported previously [33]. Instead of limiting ourselves to a narrow linear range, an appropriate nonlinear function was found to describe all data points. The estimated LOD for HRP was 6.5 pM, and the mean CV for the range above the LOD was 11%. The calibration curves for the SERS detection of HRP have the same S-shape as those for DAP, with estimated LODs of 0.067 and 0.32 pM for 733 and 1374 cm^−1^ bands, respectively. Therefore, as with the DAP measurements, the SERS detection of HRP at 733 cm^−1^ is about two orders of magnitude more sensitive compared to colorimetry, which confirms the performance of the proposed SERS-based detection system. The mean CVs for the range above the LOD were measured to be 13% and 9% for 733 and 1374 cm^−1^ bands, respectively. Notably, the SERS signal repeatability in HRP measurements was substantially higher (and comparable to the colorimetry) than in the model DAP-oPD mixtures. This could be caused by the low grade of commercially available DAP (>90%) with the unavoidable impurity of 2-hydroxy-3-aminophenazine [43]. Retrospectively, we suppose that despite higher uncertainties, the model DAP-oPD mixtures were highly useful during optimization as long as key features (calibration curve shape and SERS to colorimetry LODs ratio) were confirmed for enzymatically generated DAP.

## 3. Discussion

The measurement of HRP activity is the basis of the catalytic enhancement and detection system in an ELISA and also in some other analytical protocols. Several published papers describe the usage of an oPD substrate together with the SERS detection of a DAP product [14,15,23,27,28]. The SERS-based detection of DAP in the mixture after an HRP-catalyzed enzymatic reaction strongly depends on the conditions, and the current study addresses this topic, which has generally been neglected previously.

The AgNP colloid used in the current study is typical for SERS applications. It has been synthesized using a well-established hydroxylamine method [36] with a slightly increased hydroxylamine hydrochloride concentration (2.4 instead of 1.5 mM). This excess decreases the oxidation of AgNPs during short-term storage and also improves the resuspension of the pellet after the centrifugation due to better surface stabilization with a higher chloride concentration. Freshly synthesized colloids were used during the entire study, leaving the problem of long-term AgNP stability to later research.

**Figure A2:**
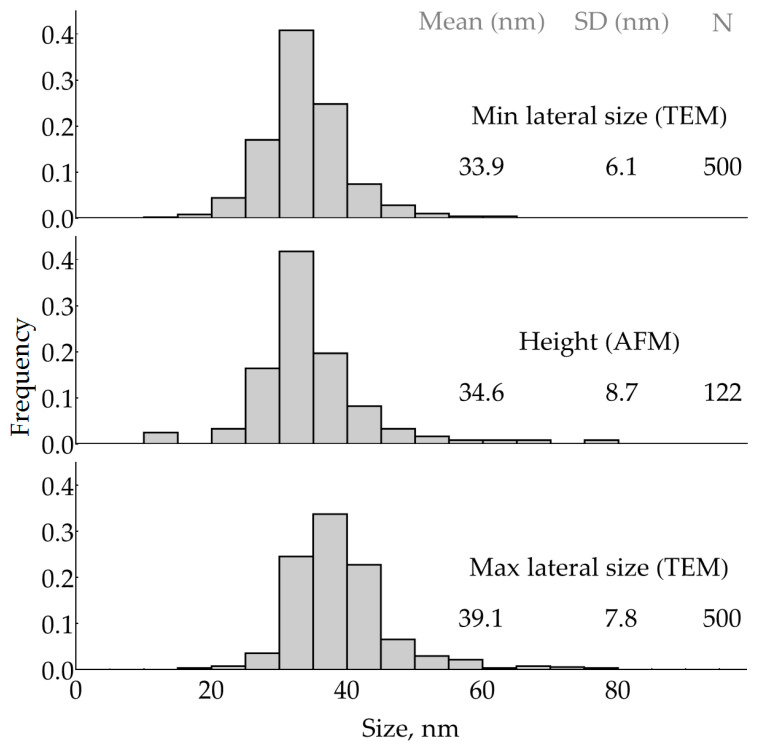
Histograms of particle size parameters measured using TEM and AFM.

**Figure A4:**
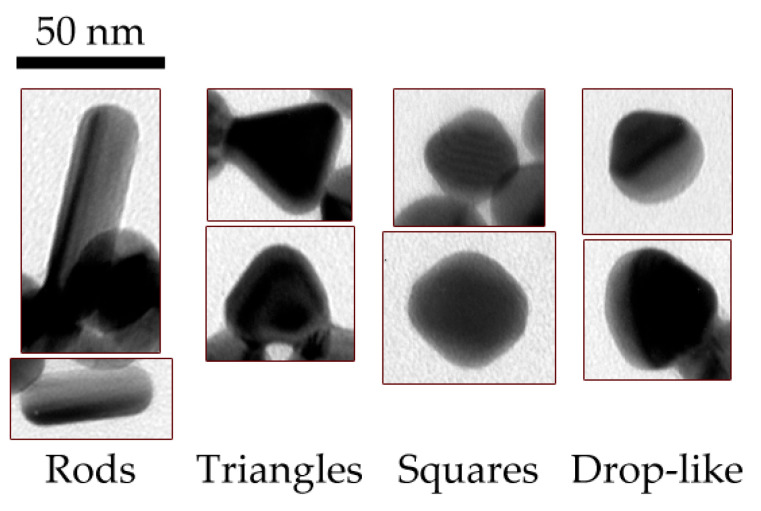
Examples for specific particle shapes in TEM images.

The particle shape is slightly prolonged with a mean aspect ratio of 1.16, which corresponds well to the value of 1.13 independently measured in our previous study [31]. About 8% of particles in total have a specific shape in TEM images: rods, triangles, squares, and a drop-like shape (Figure A4). The mean hydrodynamic spherical equivalent diameter (Figure 1b) is about 3–4 nm larger than the average microscopic size (Figure A2), which is a typical value for the thickness of two electrical double layers (one at each side of the particle) at an ionic strength of 5 mM. Therefore, most of the particles in the colloid are individual and not aggregated.

As long as fresh batches of AgNPs were used during the study, special attention was paid to their standardization. The mean particle size was standardized through the selection of synthesized colloids by the position of plasmonic band maximum in the UV–visible spectrum (Figure 1a). The medium and particle concentration of the colloid were standardized using double centrifugation and resuspension of the pellet in NaCl solution, followed by an adjustment of absorption at the plasmonic band maximum. The default NaCl concentration was chosen to be 5 mM and was later optimized to 20 mM (Figure 6). Using NTA, it was shown that double centrifugation and resuspension did not cause particle aggregation (Figure 1b). In order to preserve the desired nanoparticle concentration, the absence of their adsorption onto the laboratory plastic (Appendix G) was carefully monitored.

As mentioned previously, the SERS measurement of HRP consists of two stages: the enzymatic reaction and SERS detection of the resulting DAP. The optimization problem for this system is complicated, as these stages may require different conditions but are at the same time coupled, because the reaction mixture is passed from the first stage to the second. In order to develop a highly sensitive protocol, our attention was focused primarily on the second stage, because its performance changes hugely depending on the conditions, and the basic underlying principles were unknown. For the oPD–DAP system, both substances are commercially available. This occasion strongly simplifies the research, as it could be made with well-controlled model mixtures.

The concentration of oPD has a direct impact on the enzymatic reaction rate. At the same time, during the SERS detection, the unreacted oPD interferes with the measurement of small quantities of the produced DAP. With this contradiction in mind, first, the highest reasonable oPD concentration for the enzymatic reaction was estimated to be around 1 mM and used for further experiments.

**Figure A18:**
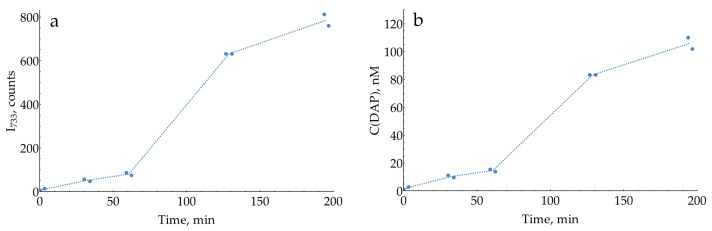
Spontaneous oPD oxidation in its 28 mM water stock stored at room temperature, measured with SERS at pH 3, 0.93 M citrate buffer at 1 mM (28-fold dilution). (**a**) Intensity of the 733 cm^−1^ band of DAP over time; (**b**) calculated DAP concentrations according to the calibration curve in Figure 7c.

The pH of SERS detection stage turned out to be the key factor affecting the sensitivity. Summarizing all the findings, DAP has a high affinity for the silver surface across the entire pH range from 0.3 up to 9.1 (Appendix C). At pH 9, AgNPs are prone to dissolution by unreacted H_2_O_2_ (Appendix D). The influence of oPD is complex. In neutral form (which exists at pH ≥ 3 according to its pK_a_ = 4.55 [44]), it exerts a moderate affinity for the silver surface (Appendix E). No other form (oPDH^+^ or oPDH_2_^2+^) was found in the SERS spectra. The neutral oPD competes with DAP for binding with silver, so at 1 mM oPD, even a relatively high DAP concentration of 500 nM is not detectable at pH > 5 (Figure 3). Second, at pH > 4, minor amounts of silver (I) present in the AgNPs facilitate the oxidation of oPD (Appendix E). The product of this process is not DAP, but most likely some intermediate on a route from oPD to DAP. The bands of this oxidation product (or products) are numerous in the SERS spectra and vary in their intensities, creating a poor background for DAP detection at pH > 4. Finally, the stock oPD contains minor quantities of DAP, both in a commercial powder and additionally formed due to spontaneous oxidation during oPD solution storage prior to use (Figure A18). This nonenzymatic DAP could deteriorate the sensitivity of the assay.

Taking into account all the listed obstacles, the sensitive SERS detection of DAP in the presence of 1 mM oPD is possible at pH ≤ 4. After the optimization of the aggregating agent concentration at each pH, a direct comparison showed the highest sensitivity at pH 3 (Figure 5). At higher pHs, DAP experiences competition with oPD for binding to the silver. At lower pHs, DAPH^+^ is partially converted into DAPH_2_^2+^ according to its pK_a_ of around 1 [41]. The coexistence of two forms of analyte typically deteriorates the SERS detection sensitivity, because the substance is split between two sets of bands. From this point of view, pH 3, being equidistant from both pK_a_s (~1 and 5.1), is a “sweet spot”, maximizing the fraction of a single form, in this case, DAPH^+^.

The optimized conditions for DAP detection rationally found in the current study are superior to those used by chance previously. In the pioneering work by Dou et al. [14], the mixture, after 20 min of enzymatic reaction at pH 5, was directly added to AgNPs. This study, although very important as the first demonstration of the principle, suffered from a poor sensitivity despite the advantage of the resonant regime with a 514.5 nm excitation. In two recent works by Fu et al. [15,27], the enzymatic reaction was stopped using sulfuric acid, and the resulting mixture was added to AgNPs. Our optimized conditions are estimated to be around 10 times more sensitive in terms of DAP detection: a five-fold difference between sulfuric acid and pH 3 (Figure 5) and an additional two-fold improvement due to modification of AgNPs with 20 mM chloride (Figure 6).

A cross-study comparison is typically not straightforward due to numerous differences in protocols. To evaluate the performance of the proposed detection system, it has been directly compared to the reference colorimetric technique. This was first performed for model mixtures of DAP with 1 mM oPD and then repeated with HRP-generated DAP. These experiments revealed an important feature of the SERS-based detection system: the calibration curve is strongly nonlinear. This is not uncommon for modern commercial assays and analytical devices with automatic signal processing. At the same time, further research would be favorable, aimed at improving the curve slope in a low concentration range. Despite these peculiarities, the proposed SERS-based detection system exhibits around two orders of magnitude lower LODs (733 cm^−1^ band) than traditional colorimetry.

The resulting LOD for HRP of 0.067 pmol/L (1.3 amol per assay) places the developed approach for SERS-based HRP measurements into a category of highly sensitive techniques, along with fluorimetry [1,2,45] and chemiluminescence [3,46,47,48]. There is obviously plenty of room for further improvements regarding the following: optimization of the enzymatic reaction stage (pH, time, temperature, buffer type, etc.), replacement of near-spherical AgNPs with anisotropic objects (rods [49], nanoplates [50], etc.), which were shown to have higher enhancement factors, and others. The proposed protocol is also compatible with classic HRP enhancement procedures such as polymeric peroxidase, tyramide signal amplification, and so on. Therefore, we suppose that this HRP–oPD system with SERS detection using AgNPs has great potential for applications in highly sensitive ELISAs.

As for the basic research, additional insights into the causes of high DAP affinity for the silver surface would be advantageous for better understanding the driving forces of the proposed analytical system. In our study, oPD provided some clues about its orientation on the silver surface thanks to the nontrivial pH dependence of SERS spectra and the presence of the broad and intense band around 983 cm^−1^ of the NH_2_ group, covalently bound to silver. On the contrary, the reasons for a high DAP affinity for silver are vague. It exhibits intense SERS spectra with AgNPs in all three forms: DAP, DAPH^+^, and DAPH_2_^2+^. Therefore, protonation does not interfere with its binding to the surface. Despite the fact that DAP and oPD both have two adjacent NH_2_ groups, the affinity of DAP for silver is much higher. See, for instance, Figure 4, for spectra at pH 6, where 100 nM DAP is visible in the presence of 100 μM oPD (1000 times the difference in concentration). The SERS spectrum of DAP also lacks the analog of the 983 cm^−1^ band of oPD for any of its forms. From these facts, our best guess is that DAP has some other orientation on the surface than oPD does. A probable faint hint is given by the concave shape of dependence in the low concentration region (Figure 7c,d), which may indicate a cooperative process of DAP oligomerization during adsorption. This hypothesis agrees with the existence of molecule ribbons in crystals of both DAP and DAPH^+^ [51,52,53], but it is hard to experimentally confirm or reject it for the surface of silver nanoparticles in a liquid. To conclude, if the proposed SERS-based HRP measurement system is to be widely used, this problem will require further research.

## 4. Materials and Methods

### 4.1. Reagents

NH_2_OH·HCl (# H9876, ≥99%), AgNO_3_ (# 209139, ≥99%), oPD (# P9029, ≥98%), DAP (# 661376, 90%), highly stabilized salt-free HRP (# P2088, RZ 2.6–3.4, 200–300 pyrogallol units/mg), NaCl (# S9625, ≥99%), bovine serum albumin (BSA, # A7030, ≥98%), trisodium citrate dihydrate (# S4641, ≥99%), anhydrous sodium acetate (# 1.06268, ≥99%), sodium dihydrogen phosphate (# 71496, ≥99%), sodium phosphate dibasic (# 1.06586, ≥99%), and (3-aminopropyl)-trimethoxysilane (# 281778, 97%) were purchased from Sigma-Aldrich. To minimize the oxidation of oPD, freshly purchased powder was stored at −20 °C instead of recommended +4 °C. Chemically pure NaOH (>99%) for AgNP synthesis and extra-pure grade H_2_SO_4_ were purchased from Chimmed, Russia. Chemically pure glacial acetic acid, extra-pure HCl (35–38 weight %), boric acid, and citric acid hydrate were purchased from Component-Reaktiv, Russia. Biotechnology-grade glycine (# Am-O167, >99%) was purchased from Helicon, Russia. Sodium tetraborate decahydrate (# 31457, 99.5%) was purchased from Fluka. All solutions were prepared using deionized water (18.2 MΩ·cm) from the MilliQ UF Plus system (Millipore, Molsheim, France).

### 4.2. Synthesis and Standardization of AgNP Colloids

AgNPs were synthesized using the hydroxylamine method by Leopold and Lendl [36], with a modified hydroxylamine hydrochloride concentration as previously described [31]. Briefly, 1 mL of 10 mM AgNO_3_ was quickly added to 9 mL of 2.56 mM hydroxylamine hydrochloride with 3.33 mM NaOH in a 15 mL polypropylene test tube (refer to Appendix G for notes) and immediately stirred using a vortex mixer. Typically, five to seven tubes were prepared at once for further selection. The mixture was stored for 1 h to complete the reaction.

Each colloid was characterized with a UV–vis absorbance spectrum (300–750 nm) in a 15-fold dilution with water using a Shimadzu UV-1800 spectrophotometer. To standardize the particle size, samples with a maximum of plasmonic band between 406.6 and 408.6 nm were selected for further use within 36 h. Prior to the SERS experiments, 1.5 mL aliquots of colloid were centrifuged for 10 min at 7000 RPM (Heraeus Sepatech Biofuge A). These conditions were chosen on this particular rotor for the highest AgNP yield as a compromise between sedimentation and resuspension completeness. The sediment was resuspended in 1.5 mL of 5 mM NaCl, followed by intensive vortex mixing. Next, the AgNP colloid was subjected to the second centrifugation, followed by resuspension in 0.75 mL of the desired NaCl concentration (2.5 to 20 mM). The absorbance at maximum (A_max_) was measured for this around a two-fold concentrate (using a 30-fold dilution in water) and adjusted by dilution in NaCl up to A_max_ = 14.25 (0.95 × 15) to standardize the particle concentration. Standardized AgNP colloids were used for SERS within 5 h to prevent their oxidation with atmospheric oxygen.

### 4.3. Determination of Mean Hydrodynamic Diameter and Total Particle Concentration of AgNPs

Some of the colloids were characterized with nanoparticle tracking analysis (NTA) using the Nanosight LM10 HS instrument (NanoSight, Amesbury, UK) in the following configuration: 405 nm, 65 mW laser unit with passive temperature readout, a high-sensitivity EMCCD-type camera, and NTA software version 2.3 build 33 software. The sample was diluted with 5 mM NaCl 15,000 or 13,000 times to reach the optimal concentration for the NTA technique. Twenty-one videos of particles’ Brownian motion (60 s each) were recorded using the following camera settings in advanced mode: shutter = 450, gain = 180, lower threshold = 455, and higher threshold = 16,380. All videos were processed in basic mode, with a detection threshold of 6 (Multi) and automatic settings for other options. The data from all repeats (at least 3600 tracks) were merged to obtain the particle size distribution, mean number-weighted hydrodynamic diameter, and total particle concentration, corrected for the dilution factor.

### 4.4. Transmission Electron Microscopy of AgNPs

One side of a nitrocellulose, carbon-coated PELCO^®^ Cu grid (Ted Pella Inc., Redding, CA, USA) was silanized by applying a 20 mkL droplet of a freshly prepared, 1% water solution of (3-aminopropyl)-trimethoxysilane (APTMS) for 2 min, followed by triple washing in water. The grid was placed into an as-synthesized AgNP colloid for 10 min for particle adsorption due to electrostatic forces between negatively charged particles and positively charged amino groups of the APTMS coating. After that, the grid was washed three times with water and dried. The control sample contained no nanoparticles, only surface silanization. Grids were examined at 80 kV with a JEM-1400 transmission electron microscope (JEOL, Tokyo, Japan) equipped with a Quemesa CCD camera (Olympus Soft Imaging Solutions, Münster, Germany). The images were manually processed in ImageJ 1.51n (National Institutes of Health, Bethesda, MD, USA) by measuring the largest and smallest dimensions for each individual particle.

### 4.5. Atomic Force Microscopy of AgNPs

A 5 × 5 mm piece of Si wafer was rinsed with water and immersed into a freshly prepared 1% water solution of APTMS for 2 min. The sample was transferred into water and vortexed to wash off the excess APTMS. This washing step was performed three times in total. The sample was purged with clean air and immersed in an as-synthesized AgNP colloid for 3 min for particle adsorption. After that, the sample was washed three times as described before and purged with clean air. The control sample contained only surface silanization and triple washing. The samples were examined with an Asylum MFP-3D-SA atomic force microscope (Asylum Research, Santa Barbara, CA, USA) in tapping mode on air, using silicon NSG03 cantilevers (NT-MDT, Moscow, Russia) with a tip curvature radius of 10 nm. The resonant frequency of the cantilever was 145.45 kHz. The force constant determined by the built-in passive thermal vibration method was 1.44 N/m. The amplitude set point was kept at 99.4–99.6% of free amplitude, and the tip velocity was set to 200 nm/s to avoid particle movement with the tip. After obtaining eight 1 × 1 μm topographic images, the absolute free amplitude of the cantilever was estimated to be 26.5 ± 1.0 nm (mean ± SD, N = 3) in the area with no particles using approach–retraction curves. In order to flatten the support level, the acquired topographic data were treated by the sequence of the following built-in procedures: (1) subtraction of the first-order plane, (2) subtraction of the first-order plane with particles masked, (3) subtraction of the mean from each line in the fast scan direction with particles masked, and (4) subtraction of the first-order polynomial from each line in the fast scan direction with particles masked. The heights of individual particles were measured using a central section method.

### 4.6. Colorimetric Estimation of HRP Effective K_M_ for oPD

The enzymatic reaction was carried out at room temperature with 80 μM of H_2_O_2_ in 100 mM sodium phosphate buffer (pH 6.0) with 5 μg/mL BSA to prevent HRP loss. The formation of DAP was followed in the kinetic regimen by measuring the absorbance at 421 nm using a UV-1800 (Shimadzu, Kyoto, Japan) spectrophotometer. After mixing the buffer concentrate, H_2_O, H_2_O_2_, and oPD in a single-use, 1 cm acrylic cuvette, the reaction was started by the addition of an HRP aliquot, followed by pipetting for 30 s and measurements of absorbance for 350 s, one point per second. During the preliminary experiments, an appropriate concentration of HRP was chosen (8 ng/mL) so that the absorbance after 5 min at 5 mM oPD did not exceed 0.25, being in a linear range for DAP colorimetric detection. Background oPD oxidation was measured for each oPD concentration the same way as the enzymatic reaction, but with the HRP aliquot replaced by buffer. The resulting pairs of kinetic curves (with HRP and without) were processed to obtain the enzymatic reaction rate (see Appendix B for details). The DAP concentration from A_421_ was calculated using an absorption coefficient of 15,560 M^−1^×cm^−1^ at pH 6. The entire dataset of the reaction rate versus the oPD concentration was fitted using the Michaelis–Menten equation in Mathematica 10.2 (Wolfram Research, Champaign, IL, USA) using the built-in NonlinearModelFit function with the weight equal to inversed data variance at each oPD concentration.

### 4.7. Preparation of Standard DAP Solutions

DAP is hardly soluble in water (70 to 100 μM depending on the temperature), so its exact solution cannot be prepared by dissolving the weighted sample. Instead, an excess amount (around 20–30 mg) of DAP was added to 12 mL of water, stirred on a vortex mixer, and sonicated for 30 min in a 40 W ultrasonic bath to create a saturated solution. After cooling to room temperature, the suspension was filtered through a 0.22 μm PES syringe filter, with the first 1 mL being withdrawn. The DAP concentration was measured colorimetrically at a five-fold dilution in water (to be in a linear range) at 421 nm, using an absorption coefficient of 15,560 M^−1^×cm^−1^. The resulting solution was stored in a dark test tube due to the light sensitivity of DAP and used the same day.

### 4.8. Procedure for SERS Measurements

As the developed analytical procedure is proposed for broad usage, all the data provided in the paper were collected using the portable spectrometer i-Raman Pro BWS475–785 H (BWTek, Plainsboro, NJ, USA) with a 785 nm excitation and 20× objective. For validation purposes during the qualitative comparison of Raman or SERS spectra, the second spectrometer, N’tegra Spectra (NT-MDT, Moscow, Russia) with a 785 nm excitation and 100× objective, was used.

An aliquot (20 μL) of sample was added to 20 μL of AgNPs (with the aggregation timer being started) and mixed by pipetting. A droplet of the mixture (20 μL) was placed on the surface of thick Al foil. The focus of the spectrometer was positioned at the top surface of the droplet and then shifted 600 μm into the liquid. Two minutes after aggregation had started, the SERS spectrum was acquired. To avoid detector saturation on samples with strongly different SERS intensities, the spectrometer setup was chosen to be as follows: 0.5 s collection time with 30 automatically averaged repeats (15 s total collection time) at 135 mW of power on the sample.

Buffers and media for different pHs were prepared from the following components: H_2_SO_4_ was diluted from concentrated acid; the pH 2 buffer was prepared from glycine and H_2_SO_4_ instead of the more common HCl to avoid the influence of chloride on the SERS signal; the pH 3 and 4 buffers were prepared from citric acid and trisodium citrate; the pH 5 buffer was prepared from acetic acid and sodium acetate; the pH 6 and 7 buffers were prepared from monosodium and disodium phosphates; and the pH 9 buffer was prepared from boric acid and sodium tetraborate.

### 4.9. Colorimetric and SERS Measurements of DAP at pH 3

In order to minimize the amount of DAP formed in the oPD solution due to spontaneous oxidation, only freshly dissolved oPD stock was used. Samples containing DAP in 1 M citrate buffer (pH 3) with 0.33 mM oPD were placed into a 96-well polystyrene plate (Greiner, # 655080) in four technical replicates (200 μL), mixed for 10 s for proper meniscus settling, and measured at 454 nm with an xMark plate reader (Bio-Rad, Tokyo, Japan). Alternatively, similar samples with lower DAP concentrations were used for the SERS measurements. Each curve was independently repeated with another oPD and DAP stocks, polystyrene plate, or AgNP batch to obtain a realistic estimate of the data variance.

### 4.10. Colorimetric and SERS Measurements of HRP

The substrate mixtures (1 mM oPD and 80 μM H_2_O_2_ in 100 mM citrate buffer at pH 6, with 5 μg/mL of BSA to prevent HRP losses at low concentrations) were prepared in test tubes with the same precaution for the oPD stock as for the DAP measurement. The reaction was initiated by the addition of HRP aliquots. After ten minutes, the reaction was stopped by the addition of twice the volume of citrate buffer 1.5 M (pH 3) and either placed into a 96-well polystyrene plate in four technical replicates (200 μL), followed by measurement with the xMark plate reader at 454 nm, or used for SERS. Each curve was independently repeated with another oPD stock, HRP dilutions, polystyrene plate, or AgNP batch to obtain a realistic estimate of the data variance.

### 4.11. Processing of Concentration Curves

The data of all concentration curves (DAP and HRP) were fitted in Mathematica 10.2 using the built-in LinearModelFit or NonlinearModelFit functions with the weight equal to inversed data variance at each DAP or HRP concentration. The limit of detection (LOD) was calculated from the calibration curve as the concentration, corresponding to the signal equal to the mean of the blank + 3 standard deviations of the blank. As long as the standard deviation strongly fluctuates for small sample sizes (N), the mean coefficient of variation (CV) for the range above the LOD was used as an estimator of assay repeatability. For each concentration, the signal SD was calculated and normalized by the difference of the mean signal and mean blank. The resulting CVs were averaged for all concentrations above the LOD.

### 4.12. Processing of Raman and SERS Spectra

For presentation purposes, the polynomial background was subtracted from each spectrum using OPUS 7.0 software (Bruker Optik GmbH, Ettlingen, Germany) through the “Baseline correction” built-in function.

To extract quantitative information about band intensities from the SERS spectra, two procedures were used in the current study (see Appendix H for additional figures).

**Figure A25:**
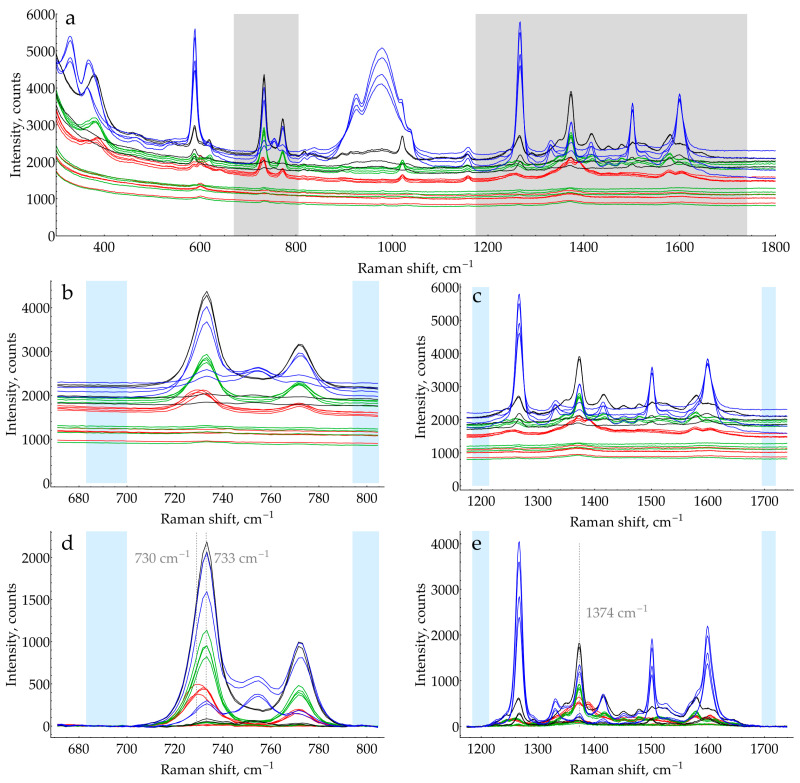
Demonstration of the first processing procedure applied to the spectra obtained under varying conditions, using a dataset from various pHs and 0 or 500 nM DAP as an example. Blue curves: pH 4; black curves: pH 3; green curves: pH 2; red curves: H_2_SO_4_. (**a**) Raw spectra; gray shading depicts two pieces used for further analysis. (**b**) The piece around the band at 733 cm^−1^; blue shading shows the ranges used for linear approximation of the baseline. (**c**) The piece around the band at 1374 cm^−1^; blue shading shows the ranges used for linear approximation of the baseline. (**d**) The same data as **b** with the baseline subtracted. (**e**) The same data as (**c**) with the baseline subtracted.

The first one contains background correction only. It has been applied to spectra obtained under varying conditions (aggregating agent concentration optimization, pH selection, and surface chloride optimization). First, two pieces were cut from each spectrum: 670–805 cm^−1^ for the 733 cm^−1^ band and 1174–1740 cm^−1^ for the 1374 cm^−1^ band (Figure A25a). Next, for each piece, two ranges for background estimation to the left and right from the desired band were chosen: 683–700 and 794–805 cm^−1^ for 733 cm^−1^ and 1174–1214 and 1695–1720 cm^−1^ for 1374 cm^−1^ (Figure A25b,c). A linear fit of the background was made for these ranges and subtracted from the cut pieces (Figure A25d,e). Finally, the intensities of the desired bands were taken at single pixels, corresponding to 733.28 and 1373.65 cm^−1^. For the curves obtained in H_2_SO_4_, the maximum intensity between 729.95 and 734.95 cm^−1^ was used, because the position of the band experienced some shifts due to the varying ratio of DAPH^+^ to DAPH_2_^2+^.

**Figure A26:**
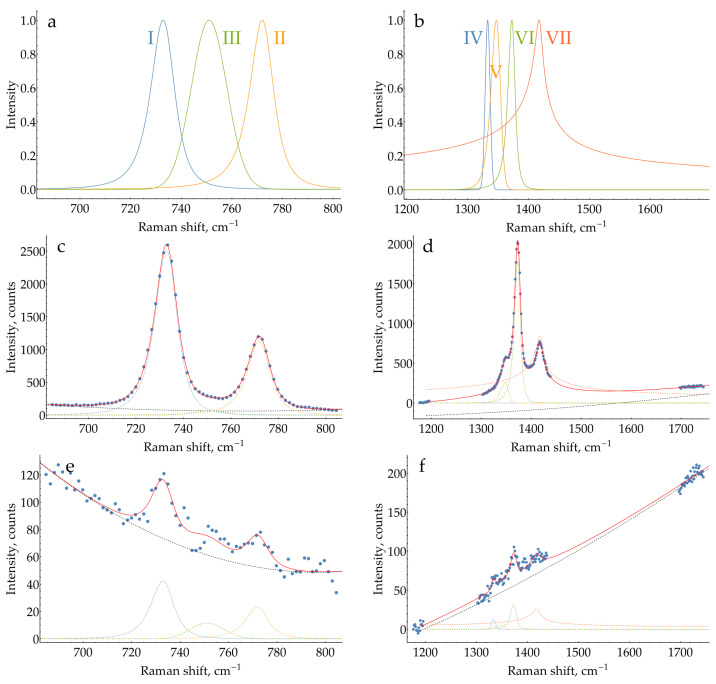
Demonstration of the second processing procedure applied to the spectra obtained in optimized conditions. (**a**) The plot of peak shapes around 733 cm^−1^. See Table A4 for equations, (**b**) The plot of peak shapes around 1374 cm^−1^. (**c**,**e**) Examples of fitting curves around 733 cm^−1^ at high and low DAP concentrations, respectively. (**d**,**f**) Examples of fitting curves around 1374 cm^−1^ at high and low DAP concentrations, respectively. Blue points represent experimental spectrum, red line represents total fit, dashed black curve represents quadratic polynomial background, and colored dashed lines represent fitted peaks.

The second procedure is more rigorous and was applied to the spectra obtained in optimized conditions only (DAP and HRP calibration curves). In this case, all the bands in the spectra have the same shape but vary in their intensities. Thus, an approach similar to the quantitative Rietveld analysis of XRD diffractograms could be exploited to reduce the noise and extract weak signals. In a preliminary stage, using several spectra at a high DAP concentration (high signal-to-noise ratio), the desired and a few neighboring bands were fitted with either a general Pearson IV peak shape or a Gaussian for weak peaks. After that, these peak shapes were fixed and normalized to have unity intensity at their maximums (see equations in Appendix H). The non-negative linear superposition of these found peak shapes, together with the quadratic polynomial background, could be used to describe any spectrum obtained in optimized conditions. In the main stage, the same pieces of spectra as for the first procedure were fit with this superposition using the built-in NonlinearModelFit function in Mathematica 10.2 (Figure A26).

### 4.13. Statistical Analysis

All calculations of statistical parameters were performed using the built-in functions of the Mathematica 10.2 package. The confidence intervals for the mean values in replicate NTA measurements were estimated using a Student t-distribution (MeanCI function) with a confidence level of 95%. A Mann–Whitney test (MannWhitneyTest function) was used for sample comparison. The differences were considered significant at *p* < 0.05.

## 5. Conclusions

The current paper is the first to describe the optimization of the SERS detection stage for the HRP–oPD–DAP system using hydroxylamine silver colloids. The pH has been shown to be the key parameter defining the sensitivity of the SERS detection of DAP in the presence of excess oPD. At a pH > 3, DAP experiences competition with oPD for binding to the silver. At a pH < 3, DAPH^+^ is partially converted into DAPH_2_^2+^, which also deteriorates the sensitivity. At an optimal mildly acidic pH of 3, a 0.93–1 M citrate buffer, and AgNPs stabilized with 20 mM chloride, an advantage of two orders of magnitude in the LODs for SERS compared to colorimetry were demonstrated for both DAP and HRP. The resulting LOD for an HRP of 0.067 pmol/L (1.3 amol per assay) makes this detection system a useful tool for the development of highly sensitive, SERS-based ELISA protocols.

## Figures and Tables

**Figure A1:**
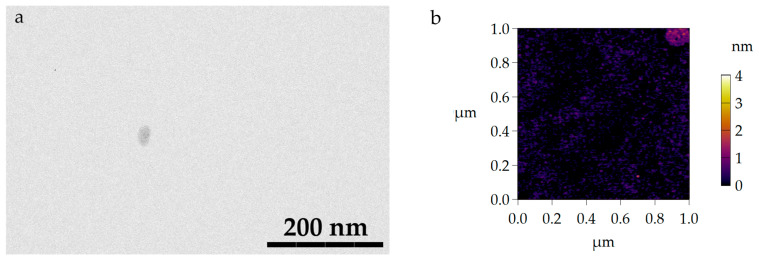
Blank samples for TEM (**a**) and AFM (**b**), corresponding to Figure 1c,d.

**Figure A3:**
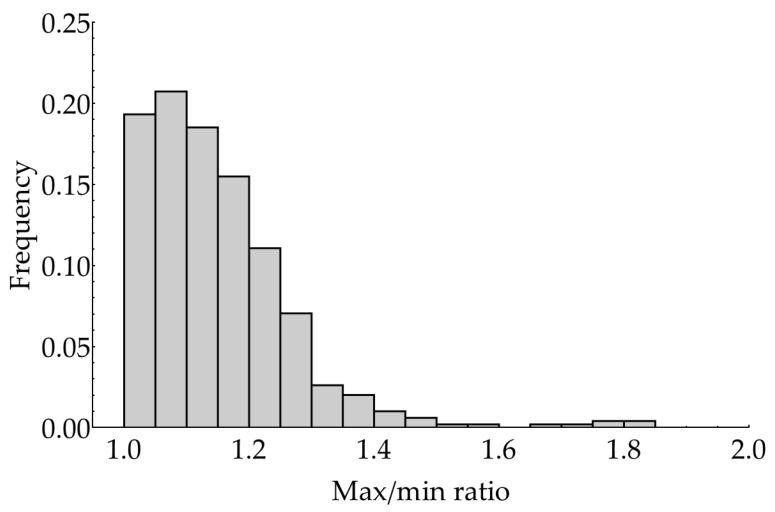
Histogram for the ratio of maximal to minimal TEM size of individual particle projections (N = 500).

**Figure A5:**
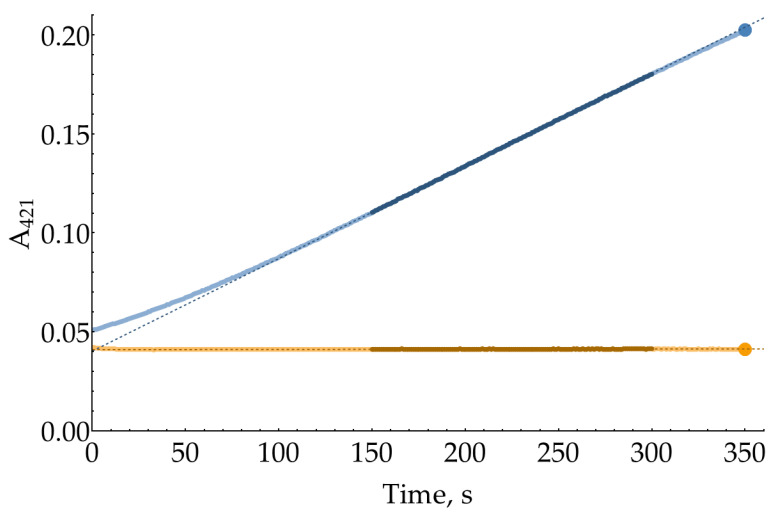
A pair of colorimetric kinetic curves with 8 ng/mL HRP, 80 μM H_2_O_2_, and 1 mM oPD in 100 mM sodium phosphate buffer (pH 6.0). The 150–300 s segments used for the slope calculation are plotted with darker markers. End points at 350 s used for the alternative rate estimation are plotted as large circle markers.

**Figure A6:**
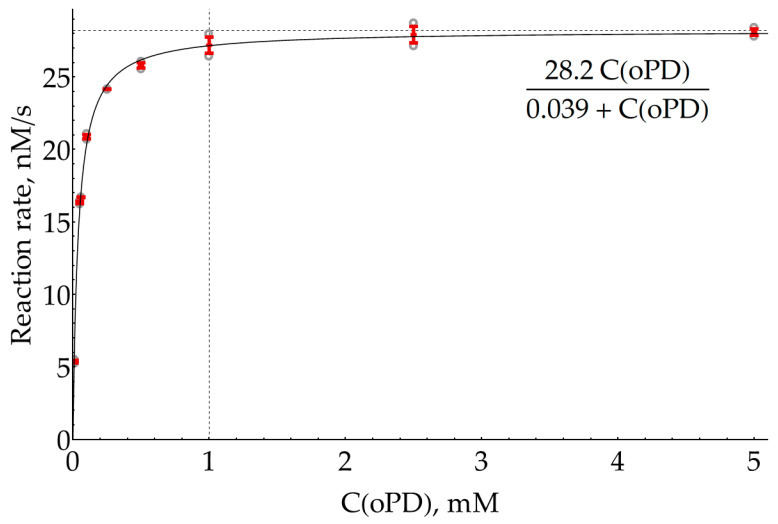
The same curve as in Figure 2, but with reaction rates calculated from the end points (350 s) of the kinetic curves. Open gray circles represent raw data (N = 2); red bars denote the mean ± standard deviation. A solid line represents the best fit.

**Figure A7:**
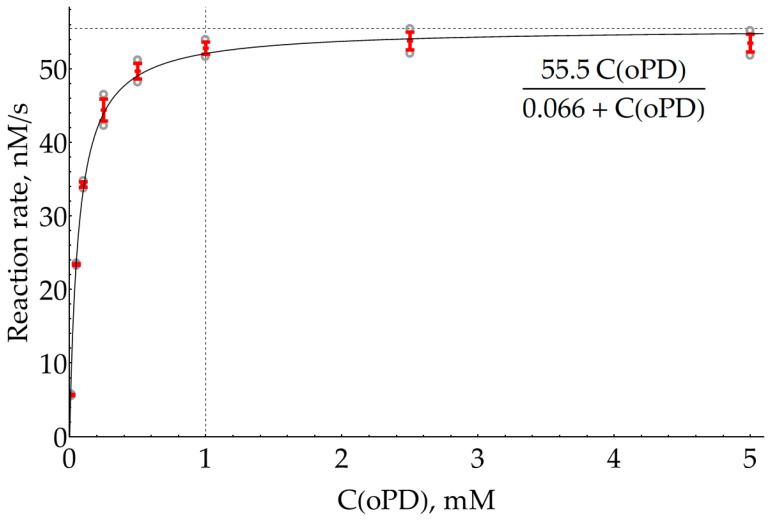
Dependence of HRP enzymatic reaction rate on oPD concentration measured colorimetrically at 421 nm in 100 mM sodium phosphate buffer (pH 6.0) with 8 ng/mL HRP and 150 μM H_2_O_2_. Reaction rates were calculated from the slopes of kinetic curves. Open gray circles represent raw data (N = 2); red bars denote the mean ± standard deviation. A solid line represents the best fit.

**Figure A8:**
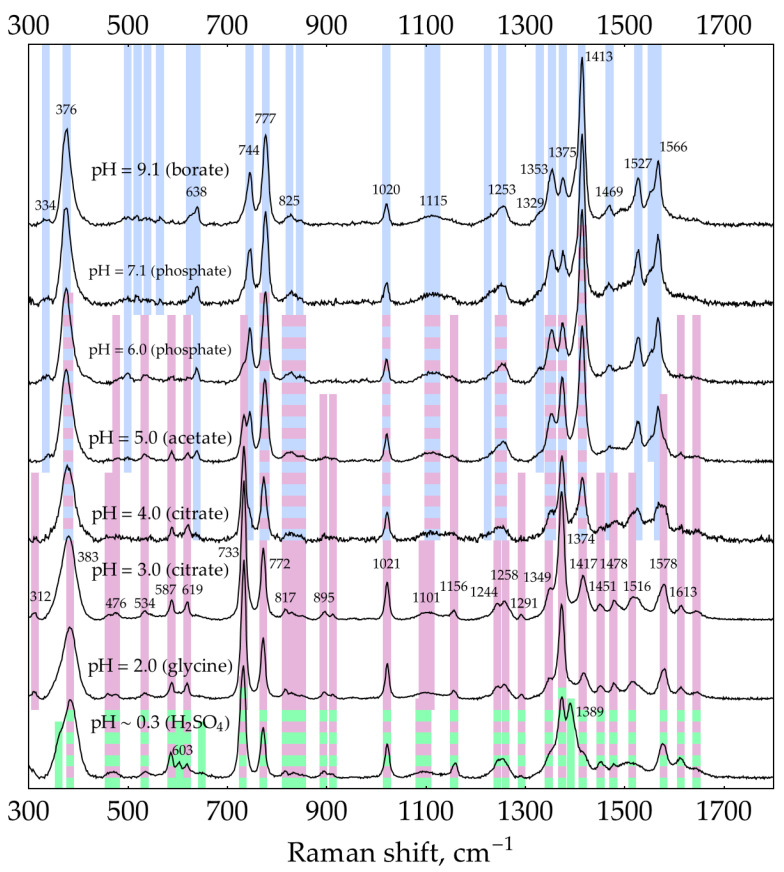
SERS spectra (135 mW, 785 nm, 15 s total accumulation time) of 500 nM DAP with AgNPs in 5 mM NaCl at different pHs. In case any weak buffer bands (with a relative intensity of a few percent) were present in the control spectrum, they have been subtracted. The polynomial baseline was also subtracted from each spectrum. Shading: blue—DAP, purple—DAPH^+^, green—DAPH_2_^2+^.

**Figure A9:**
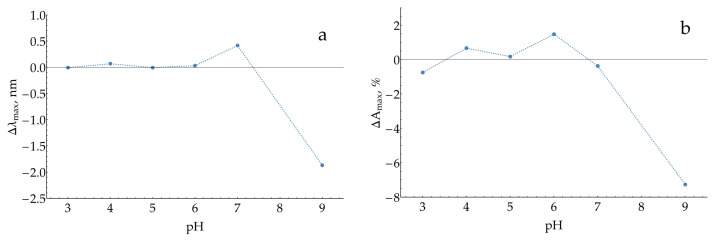
Changes in (**a**) the position of maximum and (**b**) the relative absorption at maximum for AgNPs (stabilized with 5 mM of NaCl) during 10 min of incubation with 100 μM H_2_O_2_ at different pHs.

**Figure A10:**
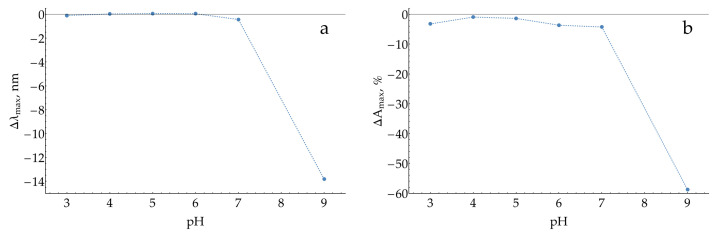
Changes in (**a**) the position of maximum and (**b**) the relative absorption at maximum for AgNPs (stabilized with 5 mM of NaCl) during 10 min of incubation with 1 mM H_2_O_2_ at different pHs.

**Figure A11:**
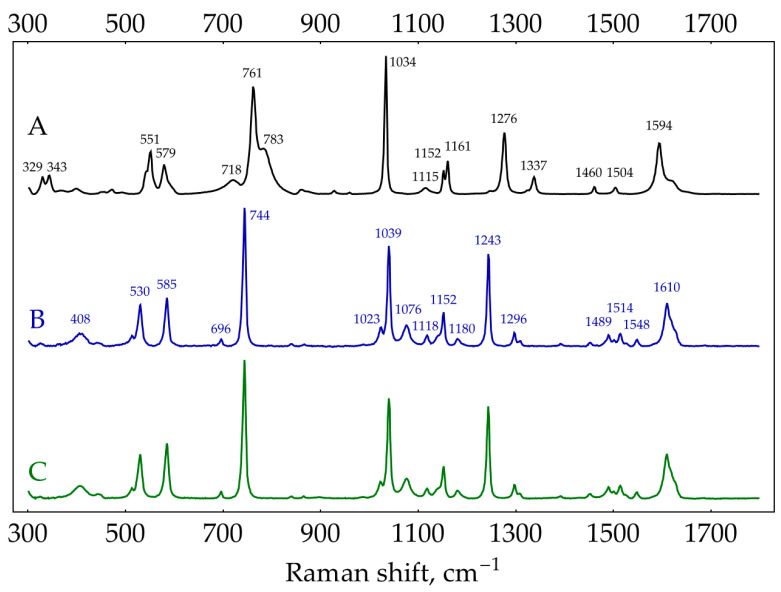
Normal Raman spectra from solids: (A) oPD Sigma P-9029 (75 mW, 785 nm, 15 s accumulation time); (B) oPD∙2HCl Alfa Aesar J60354 (240 mW, 785 nm, 15 s accumulation time); (C) synthesized oPD∙2HCl (same acquisition conditions as B).

**Figure A12:**
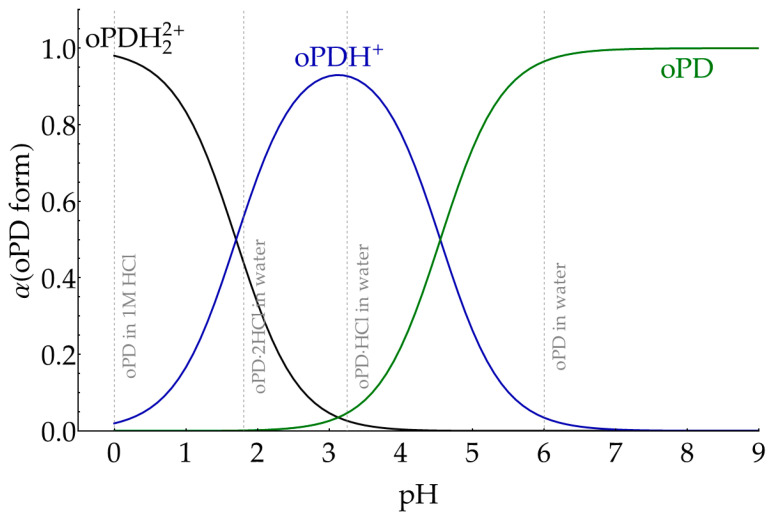
Theoretical fractions of oPD forms at different pHs, assuming unity activity coefficients.

**Figure A13:**
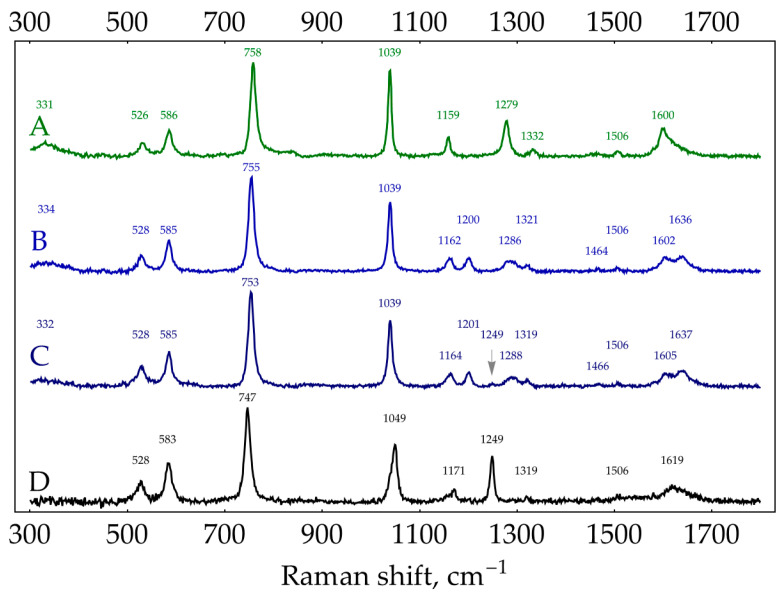
Normal Raman spectra (300 mW, 785 nm, 300 s total accumulation time, solvent background subtracted) of 27.7 mM water solutions of (A) oPD, (B) oPD∙HCl, (C) oPD∙2HCl, and (D) oPD in 1 M HCl.

**Figure A14:**
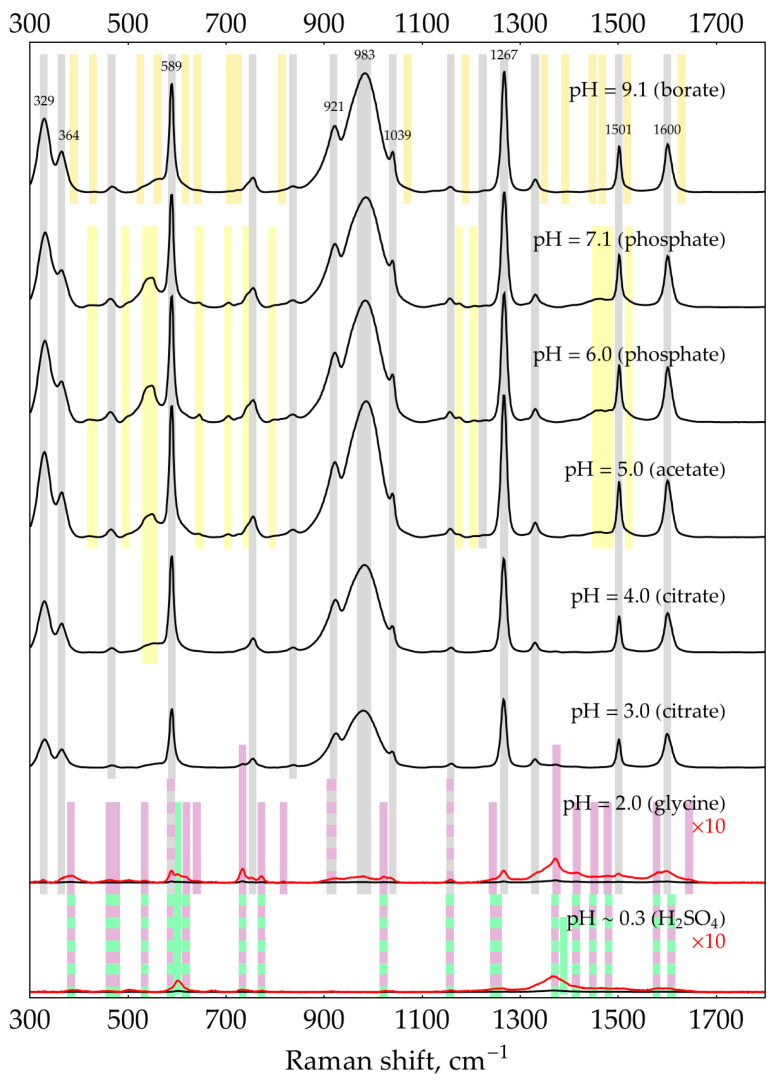
SERS spectra (135 mW, 785 nm, 15 s total accumulation time) of 10 mM oPD with AgNPs in 5 mM NaCl at different pHs. The polynomial baseline was subtracted from each spectrum. Black lines represent original spectra; red lines represent multiplied ones for visual clarity. Shading: grey—neutral oPD, orange and yellow—oxidation products of oPD, purple—DAPH^+^, green—DAPH_2_^2+^.

**Figure A15:**
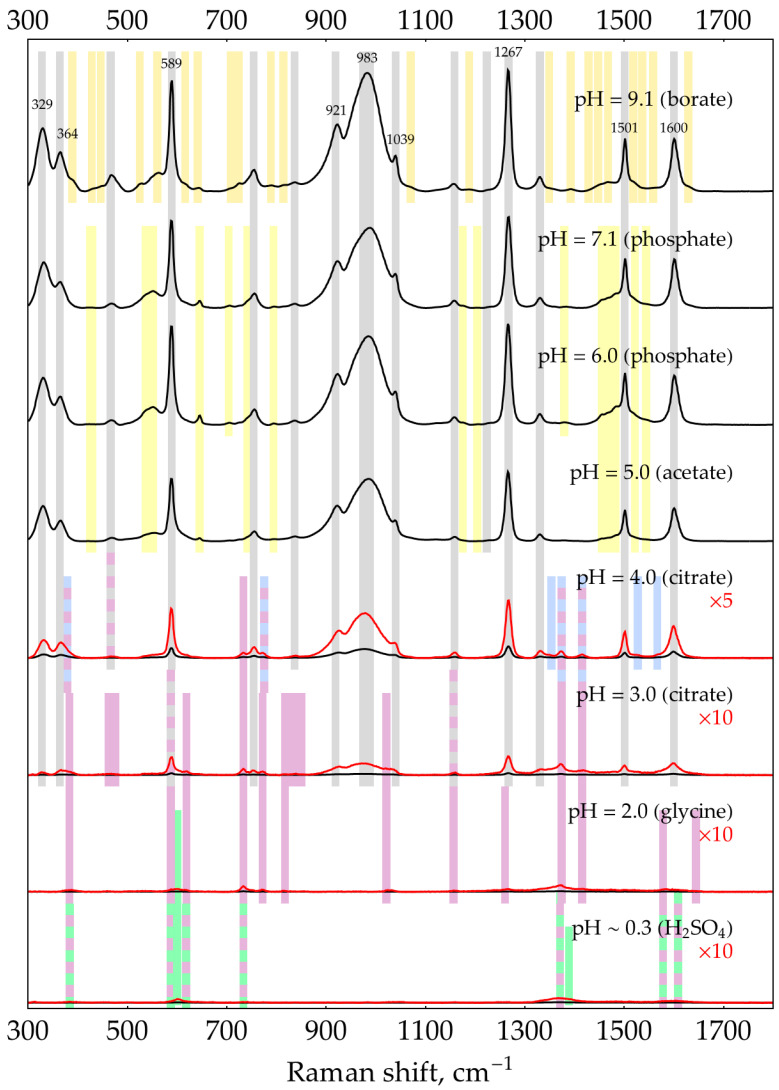
SERS spectra (135 mW, 785 nm, 15 s total accumulation time) of 1 mM oPD with AgNPs in 5 mM NaCl at different pHs. The polynomial baseline was subtracted from each spectrum. Black lines represent original spectra; red lines represent multiplied ones for visual clarity. Shading: grey—neutral oPD, orange and yellow—oxidation products of oPD, blue—DAP, purple—DAPH^+^, green—DAPH_2_^2+^.

**Figure A16:**
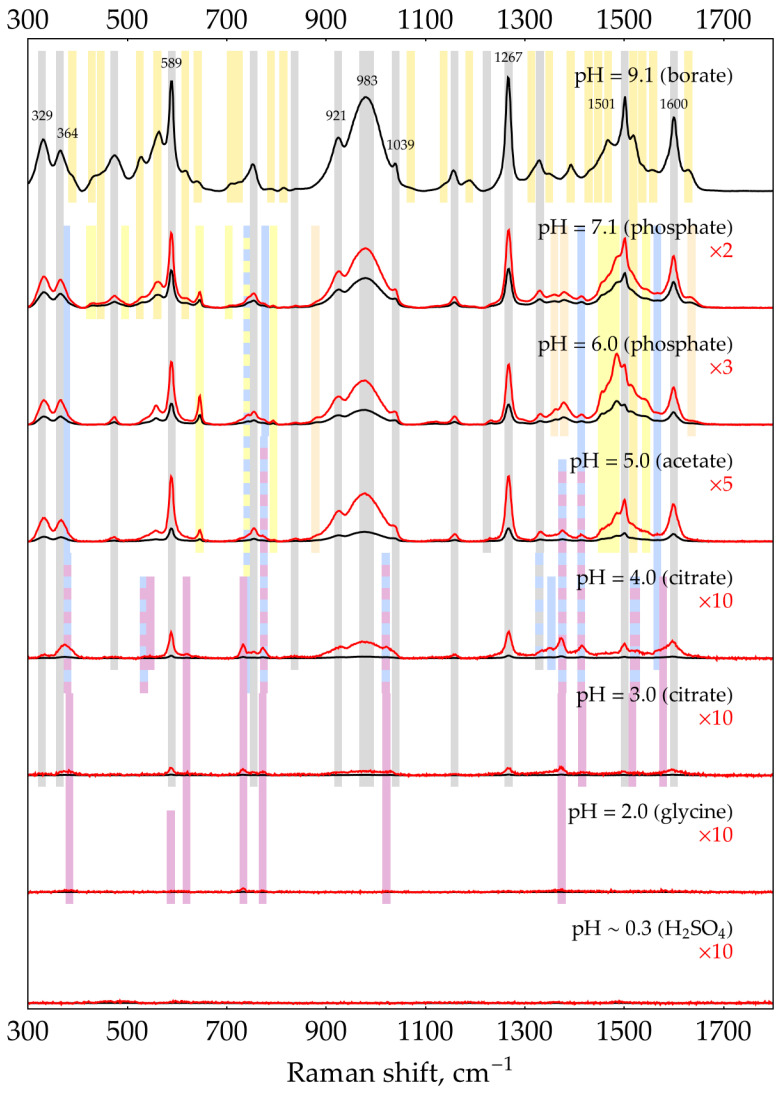
SERS spectra (135 mW, 785 nm, 15 s total accumulation time) of 0.1 mM oPD with AgNPs in 5 mM NaCl at different pHs. The polynomial baseline was subtracted from each spectrum. Black lines represent original spectra; red lines represent multiplied ones for visual clarity. Shading: grey—neutral oPD, orange and yellow—oxidation products of oPD, blue—DAP, purple—DAPH^+^.

**Figure A17:**
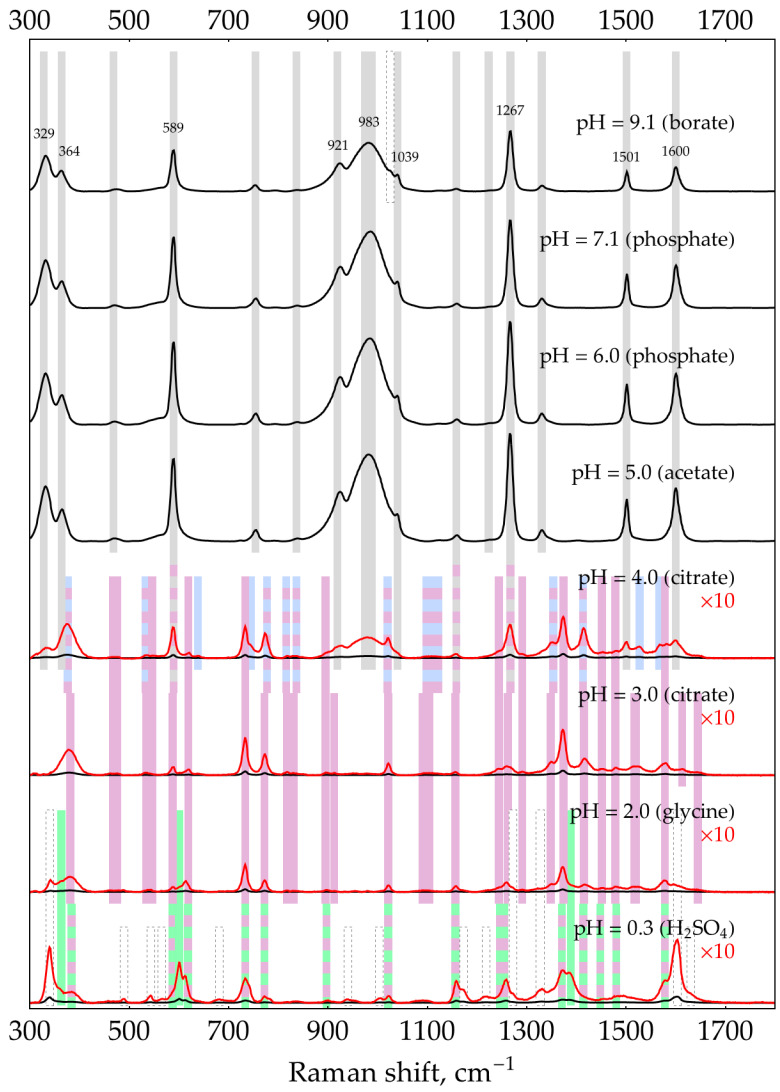
SERS spectra (135 mW, 785 nm, 15 s total accumulation time) of 0.1 mM oPD with reduced AgNPs (5 mM Cl^−^, 2 mM NH_2_OH, and 1 mM NaOH) at different pHs. The polynomial baseline was subtracted from each spectrum. Black lines represent original spectra; red lines represent multiplied ones for visual clarity. Shading: grey—neutral oPD, blue—DAP, purple—DAPH^+^, green—DAPH_2_^2+^, dashed edge—product(s) of reaction of DAP with NH_2_OH.

**Figure A19:**
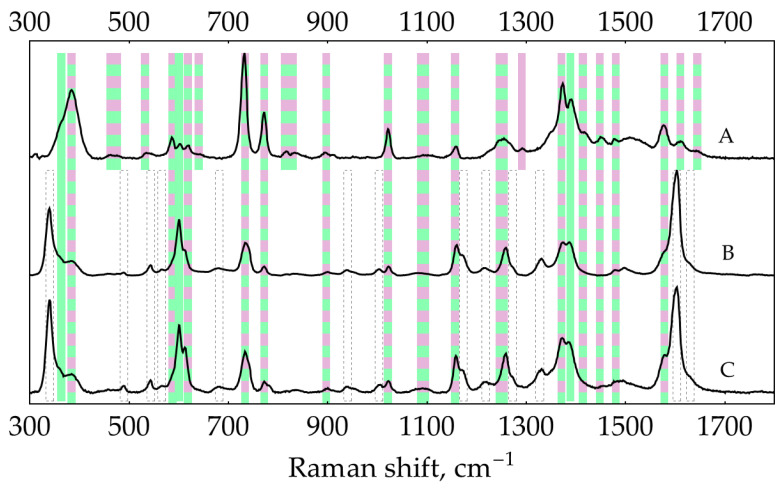
SERS spectra (135 mW, 785 nm, 15 s total accumulation time) at pH ≈ 0.3 (0.5 M H_2_SO_4_): (A) 500 nM DAP, with AgNPs in 5 mM NaCl; (B) 500 nM DAP with reduced AgNPs (5 mM Cl^−^, 2 mM NH_2_OH, and 1 mM NaOH); (C) 0.1 mM oPD with reduced AgNPs. The polynomial baseline was subtracted from each spectrum. Spectra were normalized for clarity. Shading: purple—DAPH^+^, green—DAPH_2_^2+^, dashed edge—product(s) of reaction of DAP with NH_2_OH.

**Figure A20:**
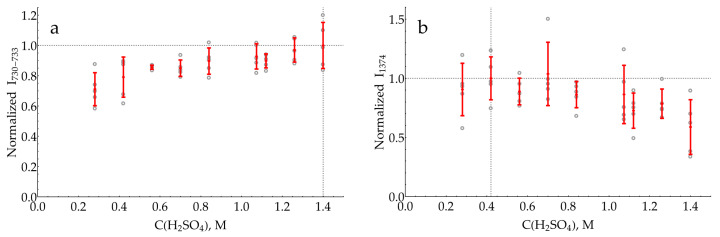
Optimization of H_2_SO_4_ concentration as an aggregating agent for AgNPs: (**a**) intensity of the band located at 730–733 cm^−1^; (**b**) intensity of the band 1374 cm^−1^. The vertical dashed line represents the chosen optimal concentration. All measured intensities were normalized by the mean intensity for this concentration. The horizontal dashed line represents 1 (100%) for visual comparison. Open gray circles represent data repeats (N = 5); red bars denote the mean ± standard deviation.

**Figure A21:**
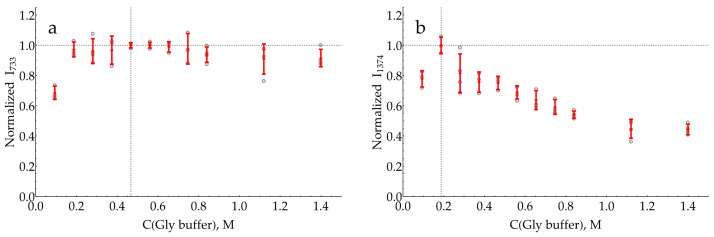
Optimization of pH 2 glycine buffer concentration as an aggregating agent for AgNPs: (**a**) intensity of the band 733 cm^−1^; (**b**) intensity of the band 1374 cm^−1^. The vertical dashed line represents the chosen optimal concentration. All measured intensities were normalized by the mean intensity for this concentration. The horizontal dashed line represents 1 (100%) for visual comparison. Open gray circles represent data repeats (N = 4); red bars denote the mean ± standard deviation.

**Figure A22:**
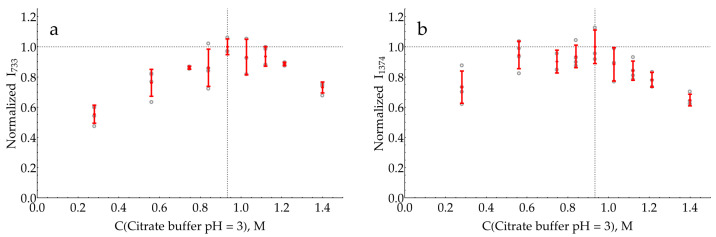
Optimization of pH 3 citrate buffer concentration as an aggregating agent for AgNPs: (**a**) intensity of the band 733 cm^−1^; (**b**) intensity of the band 1374 cm^−1^. The vertical dashed line represents the chosen optimal concentration. All measured intensities were normalized by the mean intensity for this concentration. The horizontal dashed line represents 1 (100%) for visual comparison. Open gray circles represent data repeats (N = 4); red bars denote the mean ± standard deviation.

**Figure A23:**
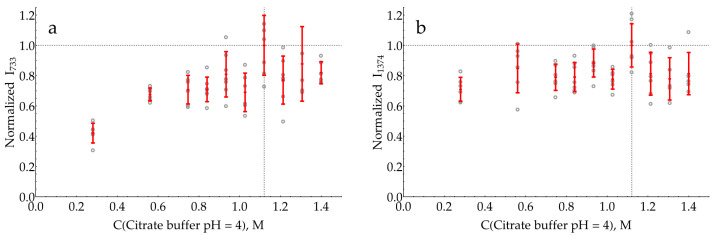
Optimization of pH 4 citrate buffer concentration as an aggregating agent for AgNPs: (**a**) intensity of the band 733 cm^−1^; (**b**) intensity of the band 1374 cm^−1^. The vertical dashed line represents the chosen optimal concentration. All measured intensities were normalized by the mean intensity for this concentration. The horizontal dashed line represents 1 (100%) for visual comparison. Open gray circles represent data repeats (N = 6); red bars denote the mean ± standard deviation.

**Figure A24:**
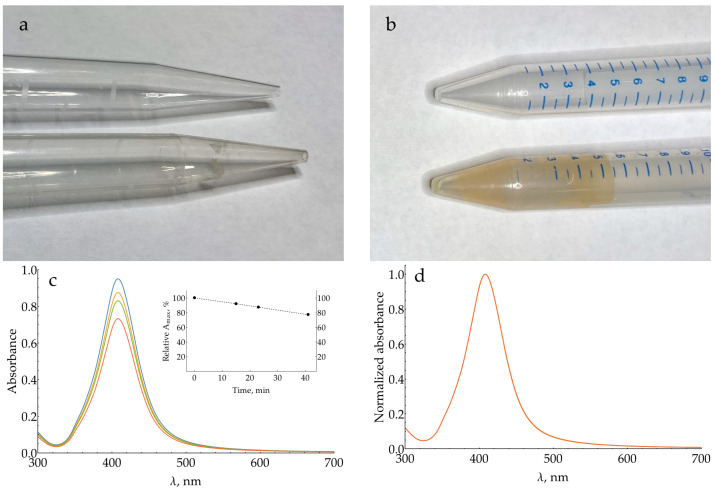
Demonstration of AgNP losses due to adsorption on polypropylene plastic. (**a**) SSI 5 mL tips: new one (top) and one used once to dose AgNPs in 5 mM NaCl (bottom). Note the black patches of adsorbed AgNPs on the inner surface of the bottom tip. (**b**) Two different lots of Greiner 15 mL test tubes used for overnight storage of AgNPs in reaction medium. The tube from the bottom lot is not appropriate for AgNP synthesis or manipulation. (**c**) UV–vis absorbance of AgNPs in 20 mM NaCl in a test tube from a bad lot with constant mixing. In 41 min, 23% of particles were lost. (**d**) The same data as (**c**), normalized by A_max_. Note that all four spectra are identical. Nothing occurs to the AgNPs except losses to adsorption.

## Data Availability

All the relevant data are provided in the main text or appendices.

## Abbreviations

AgNPs	silver nanoparticles
CV	coefficient of variation
DAP	2,3-diaminophenazine
DAPH^+^	protonated 2,3-diaminophenazine
DAPH_2_^2+^	doubly protonated 2,3-diaminophenazine
ELISA	enzyme-linked immunosorbent assay
HRP	horseradish peroxidase
LOD	limit of detection
NTA	nanoparticle tracking analysis
oPD	*o*-phenylenediamine
oPDH^+^	protonated *o*-phenylenediamine
oPDH_2_^2+^	doubly protonated *o*-phenylenediamine
SERS	surface-enhanced Raman scattering

## Appendix A

This appendix contains additional information about the particle shapes measured using TEM and AFM.

Figure A1 shows blank samples for TEM and AFM, containing only silanized supports and no AgNPs applied (corresponding to Figure 1c,d). The TEM images indeed contain no particles, but rather, scarce objects with poor contrast, typically attributed to defects of the nitrocellulose film. The AFM topography contains scarce objects with heights not exceeding 2.3 nm.

Figure A2 provides the histogram data for the minimal and maximal lateral sizes of the individual nanoparticles measured using TEM as well as the particle heights measured using AFM. Figure A3 depicts a histogram for the ratio of maximal to minimal TEM lateral size.

For the complete characterization of the particle shape, frequencies for specific shapes (see examples in Figure A4) were calculated from the TEM data. Rods occurred in 8/577 (1.4%) cases, triangles with rounded corners in 12/577 (2.1%) cases, squares with rounded corners in 15/577 (2.6%) cases, and drop-like particles in 13/577 (2.3%) cases. Together, these specific shapes represent around 8% of the particles by number.

## Appendix B

This appendix contains additional information about the measurement of the HRP effective Michaelis constant for oPD. The colorimetric kinetic curves of DAP formation showed a deviation from linearity both within short time periods (below 150 s) and after 300 s (Figure A5). We attribute deviations at the beginning of the curve to the formation of intermediates, which have been previously reported for this reaction [30,54].

For Figure 1 and the estimation of an effective K_M_ for oPD in Section 2.2, the enzymatic reaction rate was calculated as the difference between the slopes for enzymatic and nonenzymatic reactions using the linear segments between 150 and 300 s. Alternatively, the enzymatic reaction rate may be calculated using the difference between end points of the curves (350 s). This type of processing (Figure A6) results in a 95% confidence interval for K_M_ of 39 ± 3 μM, which is very close to the value calculated from the slopes.

It is also worth mentioning that 80 μM of H_2_O_2_ is quite a low concentration, used in [35] to minimize the background oxidation of oPD. In case a slightly higher peroxide concentration would be required to increase the enzymatic reaction rate, the effective Michaelis constant was also measured at 150 μM of H_2_O_2_ (Figure A7). As expected from the Ping-pong Bi–Bi mechanism for HRP, the effective K_M_ for oPD increased up to 66 ± 10 μM. However, 1 mM of oPD is still around 15 × K_M_ and may be considered a reasonable upper limit.

## Appendix C

Previously, we reported that DAP has intense SERS spectra with hydroxylamine silver colloids in a broad range of pHs from 2 to 9 [31]. In this appendix, we confirm our previous findings and extend the range of pHs down to strongly acidic conditions. We provide all the DAP SERS spectra (Figure A8) to serve as a reference for comparison throughout the paper. All spectra were recorded at a buffer concentration of 0.5 M except for borate at pH 9.1, used at a concentration of 0.32 M due to the limited solubility of borax.

- At pHs from 7 to 9, the SERS spectra correspond to neutral DAP and agree well with the normal Raman spectrum of solid DAP [31]. The neutral form of DAP is shaded blue in Figure A8. The table of shifts is provided in [31].
- At pHs from 4 to 6, a transition at around pH = 5 occurs in agreement with DAPH^+^ pK_a_ = 5.1 [31,41].
- At pHs from 2 to 3, the dominant DAP form is DAPH^+^, with ring nitrogen being protonated as evidenced by XRD [52,53] instead of the NH_2_ group as proposed by Brown and coauthors [41]. The SERS spectrum of DAPH^+^ corresponds well with the normal Raman spectrum of crystalline DAP×HCl [31]. Notably, the bands at 733 and 773 cm^−1^ are strongly enhanced in the SERS spectrum compared to a normal Raman spectrum. DAPH^+^ is shaded purple in Figure A8. The table of shifts is provided in [31].
- At pHs from 0.3 to 2, the second protonation occurs in agreement with DAPH_2_^2+^ pK_a_ of around 1 [41]. According to the law of acid–base equilibrium, a relatively pure (>99%) acidic form appears at pH = pK_a_–2 ≈ −1. Thus, the pure spectrum of DAPH_2_^2+^ may be acquired in a concentrated acid only, which is corrosive to AgNPs. As a result, most of the bands present in the spectrum at pH ~0.3 may correspond to either DAPH^+^ or DAPH_2_^2+^. However, two unique bands at 603 and 1389 cm^−1^ as well as the shoulder at around 439 cm^−1^ could be univocally assigned to DAPH_2_^2+^. They are shaded green in Figure A8.

## Appendix D

This appendix is devoted to the issue of potential AgNP dissolution with unreacted H_2_O_2_ present in the mixtures after the enzymatic reaction stage. This process was followed spectrophotometrically by measuring the parameters of the AgNP plasmonic absorption band. The position of maximum (λ_max_) is a measure of the mean particle size, and the absorption at maximum (A_max_) is related to the particle concentration.

First, for each buffer in our pH range, the maximum buffer concentration that did not cause AgNP aggregation was estimated using the absence of changes in A_max_ and long-wavelength absorption (A_500_ and A_600_) as criteria. In all subsequent experiments, lower buffer concentrations (see Table A1) were used to ensure that no particle aggregation occurred.

**Table A1:** Buffers used to test the AgNP dissolution with H_2_O_2_.

pH	Buffer Type and Concentration
3	citrate 30 mM
4	acetate 30 mM
5	acetate 20 mM
6	MES 30 mM
7	HEPES 10 mM
9	borate 50 mM

Next, for each pH, pairs of samples were compared after 10 min of incubation: (1) AgNPs in the buffer versus (2) AgNPs in the buffer with 100 μM of H_2_O_2_ (Figure A9). At pH 9, a considerable decrease in both the λ_max_ and A_max_ was observed, which indicates the dissolution of nanoparticles.

To verify our findings, the experiment was repeated with 1 mM of H_2_O_2_ (Figure A10). The observed effect was found to be dose dependent, as expected for H_2_O_2_-induced AgNP dissolution.

Thus, we may conclude that a considerable dissolution of AgNPs may occur at pH 9 with the assay-relevant H_2_O_2_ concentration of 100 μM, which may interfere with enzymatic product SERS detection.

## Appendix E

*o*-Phenylenediamine is a substrate of the peroxidase reaction, which resulted in the formation of DAP, the key molecule in the present study. Thus, the mixtures to be analyzed by SERS always contain small amounts of DAP and an excess of oPD. Both molecules compete for binding with the surface of silver nanoparticles. A deep understanding of the oPD SERS behavior facilitates rational improvements in DAP detection procedures in such mixtures. Here, we discuss our findings together with published data to create a holistic picture of this behavior.

### Appendix E.1. Normal Raman Spectra of oPD

First, normal Raman spectra were recorded for the crystalline oPD (Sigma P-9029) and oPD dihydrochloride (oPD∙2HCl, 98%+, Alfa Aesar J60354) with a 785 nm excitation (Figure A11). The spectrum of oPD agrees well with previously reported data obtained with a 514.5 or 647.1 nm excitation [37], 976 nm FT-Raman [55], and 532 nm excitation [56]. Our spectrum of commercial oPD∙2HCl substantially differs from the one synthesized by Koh et al. [37]. To clarify this discrepancy, we have synthesized oPD∙2HCl from oPD according to [57]. Briefly, excess oPD was added to concentrated HCl (density 1.19 g/cm^3^) and heated to 95 °C for at least 30 min. Undissolved oPD was separated using a short centrifugation (1 min, 3000 rpm). A slightly pink-colored supernatant was cooled to +4 °C. White crystals were separated, washed with cold concentrated HCl, and dried under vacuum (<1 mTorr) for 1.5 h. The normal Raman spectrum of this oPD∙2HCl was identical to the commercial Alfa Aesar reagent (Figure A11). Thus, there must be some issue with the spectrum of solid oPD∙2HCl reported in [37]. This most likely corresponds to hydrochloride oPD∙HCl rather than dihydrochloride oPD∙2HCl.

Providing trustworthy information about the vibrational bands of particular molecules, Raman spectra from solid substances may not be straightforward to compare with SERS spectra due to several complications:

- Raman spectra from solids may contain lattice modes;
- Some shifts in band positions and intensities may occur due to interactions between molecules in the crystal;
- For anisotropic crystals, slight shifts in the band positions and possibly large changes in the relative intensities may occur due to the preferred crystal orientation [58].

The Raman spectrum from the water solution, typically with a lower signal-to-noise ratio compared to one from a solid, nevertheless provides additional insights into the vibrational bands of the molecule in the well-separated and solvent-surrounded state. On the other hand, the interpretation of solution Raman spectra should be made under the consideration of equilibriums between various forms of the target molecule (e.g., acid–base equilibrium).

Thus, prior to the assignment of solution spectra, we have theoretically estimated which forms of oPD preferably exist in different water solutions. According to [44], oPDH_2_^2+^ has an apparent pK_a,1_ = 1.70 and an apparent pK_a,2_ = 4.55. Under the simplest model, which assigns all activity coefficients to 1, we calculated the fraction of each oPD form in solution at different pHs (Figure A12) as well as pHs for the oPD∙HCl and oPD∙2HCl solutions (Table A2). The pH of oPD in water is assumed to be around 6 due to atmospheric CO_2_.

**Table A2:** Calculated pHs and fractions of oPD forms in different solutions at total oPD concentration of 27.7 mM.

System	pH	α (oPDH_2_^2+^), %	α (oPDH^+^), %	α (oPD), %
oPD in 1 M HCl	0	98	2	0
oPD∙2HCl in water	1.8	44	56	0
oPD∙HCl in water	3.3	2.5	93	4.5
oPD in water	≈6	0	3	97

According to the provided estimations, we expect that the spectrum of oPD in water is dominated by the neutral form, the spectrum of oPD∙HCl in water contains mostly oPDH^+^ bands, the spectrum of oPD∙2HCl in water corresponds to the mixture of oPDH_2_^2+^ and oPDH^+^, and the spectrum of oPD in 1 M HCl is dominated by oPDH_2_^2+^.

The following four solutions were prepared: (1) 27.7 mM of oPD in water, (2) 27.7 mM of oPD + 27.7 mM HCl, (3) 27.7 mM of oPD + 55.4 mM HCl, and (4) 27.7 mM of oPD in 1 M HCl. Ten Raman spectra with a 30 s collection time each were acquired at 785 nm and 300 mW. Each set of spectra was averaged and subtracted by averaged blanks (water, 27.7 mM HCl, 55.4 mM HCl, and 1 M HCl, respectively). The resulting spectra are shown in Figure A13.

Normal Raman spectra of solid oPD and an oPD solution show adequate correspondence (neutral oPD) as well as solid oPD∙2HCl and oPD solution in 1 M HCl (oPDH_2_^2+^). The water solution spectra of both oPD∙HCl and oPD∙2HCl at 27.7 mM closely resemble each other, as does the spectrum of solid “oPD∙2HCl” reported by Koh et al. [37]. Although the oPD∙2HCl solution spectrum should be a mixture of two forms, oPDH_2_^2+^ seemingly has approximately 30% lower Raman cross-section compared to oPDH^+^ (the absolute intensity of a 755 cm^−1^ band of oPDH^+^ was 312 counts versus 209 counts for a 747 cm^−1^ band of oPDH_2_^2+^ at identical concentrations and spectrometer setups). Thus, the presence of oPDH_2_^2+^ in the water solution spectrum of oPD∙2HCl resulted in a weak band at 1249 cm^−1^ (marked with an arrow in Figure A13, spectrum C) and a slight shift of the 755 cm^−1^ band to 753 cm^−1^.

Conclusions on the Raman spectra of oPD:

- The Raman spectra of solid oPD (spectrum A in Figure A11, [37,55,56]) and the water solution of oPD (spectrum A in Figure A13) could be used as references for the neutral form of oPD.
- The Raman spectrum of the oPD∙HCl water solution (spectrum B in Figure A13) could be used as a reference for the oPDH^+^ form.
- The Raman spectra of solid oPD∙2HCl (spectra B and C in Figure A11) and the solution of oPD in 1 M HCl (spectrum D in Figure A13) could be used as references for the doubly protonated oPDH_2_^2+^ form.

### Appendix E.2. SERS Spectra of oPD with AgNPs

The SERS spectra of oPD with AgNPs are quite complicated and probably deserve a dedicated paper. However, in this subsection, we will try to understand them just enough for rational analytical applications of oPD and DAP SERS. These data might also be used as a starting point for further fundamental research on oPD SERS.

First, oPD SERS spectra were acquired at a pH range from 0.3 to 9.1 and three different oPD concentrations: 0.1, 1, and 10 mM. AgNPs stabilized by 5 mM NaCl were used. The concentrations of all buffers/media were 0.5 M, except for the 0.32 M borate with a pH of 9.1 due to the limited solubility of borax. Silver colloid was mixed with oPD in a buffer in a 1:1 ratio and incubated for 2 min for particle aggregation, followed by the acquisition of SERS spectra from a 20 μL droplet. For oPD concentrations of 1 and 10 mM, bands of buffers/media were negligible; for 0.1 mM, a very weak spectrum of buffer/medium was subtracted from the oPD spectrum for clarity. The acquired SERS spectra of oPD are provided in Figure A14, Figure A15 and Figure A16. This set of spectra is complicated, showing both pH and concentration dependence with multiple species involved.

In order to rationalize all these spectra, two more pieces of data are required. The first is a set of spectra for DAP at different pHs (Figure A8). The second piece is the set of SERS spectra for 0.1 mM oPD, similar to Figure A16 but obtained with AgNPs in the media, containing 3 mM NaCl, 2 mM NH_2_OH∙HCl, and 3 mM NaOH (Figure A17). The total Cl^−^ concentration is 5 mM (3 from NaCl and 2 from NH_2_OH∙HCl). Hydroxylamine is added as a strong reducing agent. For the desired result, the pH should be alkaline. Two mM of NaOH out of three is used to neutralize the HCl from hydroxylamine hydrochloride, and 1 mM remains, creating a pH > 10. Thus, under these conditions, all the silver is reduced to metal, even in the presence of atmospheric oxygen. Unfortunately, hydroxylamine is unstable and slowly decomposes. These “reduced silver colloids” were used not more than 1.5 h after the preparation. It should also be mentioned that 1 mM of NaOH is negligible compared to the 0.5/0.32 M of buffer/medium used for aggregation and does not affect the pH upon SERS spectra acquisition.

Band set A corresponds to neutral oPD. It is shaded gray in Figure A14, Figure A15, Figure A16 and Figure A17 and spans from pH 9 down to pH 2–3, depending on the oPD concentration. It is the only spectrum present on “reduced silver colloid” at a neutral pH of 5–7 (Figure A13). It fully corresponds to the normal Raman spectrum of neutral oPD (Table A3) and the previously reported SERS spectrum of 100 or 10 μM oPD with gold nanoparticles [37], except for the broad and intense band at 983 cm^−1^. This band is attributed to the formation of a covalent bond between a nitrogen atom of a neutral amino group and the silver atom [37] via the free electron pair of nitrogen. The most convincing evidence of this assignment is that this band is strongly shifted upon deuteration [37]. With gold nanoparticles, this band is located at 1020 cm^−1^ [37]. It should be noted that this pattern (a broad SERS band with AgNPs around 950–985 cm^−1^ absent in normal Raman) also holds for some other amino-substituted benzenes: *o*- and *p*-aminobenzoic acids [59,60,61], aniline, and all three isomers (2-, 3-, and 4-) of aminobiphenyl [62]. A debate exists as to whether this band should be assigned to the NH_2_ rocking based on the characteristic Raman shift for this mode [37] or to the wagging mode, which is shifted due to strong interaction with metal [63,64]. Nevertheless, this open theoretical question is not important for our analytical purposes, as both sides agree that this SERS band occurs due to the covalent binding of the NH_2_ group to the metal surface.

**Table A3:** Band positions in cm^−1^ and relative intensities (%) of normal Raman and SERS spectra of neutral oPD.

Raman 785 nm, Solid oPD	Raman 785 nm, 27.7 mM oPD in Water	SERS 785 nm of oPD with AgNPs, Present Work	SERS 647 nm of oPD with a Gold Colloid at pH = 5 [37]	SERS 647 nm of oPD with a Silver Colloid at pH = 5 [37] *
329 (13)	331b (16)	329 (61)	350	330
343 (13)				
369 (2)	Shoulder of 331	364 (34)	382	362
398 (4)				
453 (2)	449 (2)			
472 (3)		467 (4)	480	476
492 (1)				
543 (16)	526 (14)	Shoulder of 589		
551 (31)				
579 (21)	586 (28)	589 (90)	586	588
718b (10)				
761 (78)	758 (100)	753 (12)	752	756
783b (33)	Shoulder of 758			
	835 (5)	836 (5)		
861 (3)				
928 (2)	908b (2)	921 (55)	924	922
959 (1)				
		983b (98)	1020b	988b
1034 (100)	1039 (91)	1039 (33)	1040	1040
1115 (4)				
1152 (17)	1159 (20)	1157 (4)	1160	1156
1161 (24)				
1246 (2)	1236 (2)	1225 (2)		
1276 (44)	1279 (38)	1267 (100)	1262	1268
1324 (3)	1332 (8)	1330 (11)		1324
1337 (12)			1340	1348
1460 (5)	1461b (3)			
1504 (5)	1506 (6)	1501 (38)	1500	1498
1594 (37)	1600 (30)	1600 (40)	1596	1598
1618 (10)	Shoulder of 1600			1628
1652 (1)				

* Only the bands of oPD are provided, but not its oxidation products; b: represents a broad band.

Unlike DAP (see Appendix C), whose SERS spectrum changes from neutral to the protonated form between pH 4 and 6 in full agreement with its pK_a_ of 5.1, the SERS band positions of oPD do not change from pH 9 down to at least pH 3 (at low concentrations) or even 2 (at 1 and 10 mM), despite its pK_a_ of 4.55 [44]. The overall intensity of the bands, however, strongly decreases in acidic conditions. This finding implies that only a neutral form of oPD has some affinity for the silver surface, most likely because both NH_2_ groups bind to Ag. We have not performed any molecular modeling as it is far beyond the analytical scope of the present paper, but general geometric considerations do not contradict this hypothesis. The calculated distance between two nitrogen atoms in oPD is 2.82 or 2.76 Å based on the optimized geometry reported in [65] or [56], while the doubled atomic radius of silver is 2.88 Å. Thus, when the first NH_2_ group binds to the surface silver atom, the second nitrogen ends up right above the adjacent silver atom.

Band set B (marked with three types of yellowish and orange shading in Figure A14, Figure A15 and Figure A16) is present at pHs from 5 to 9. As these bands are totally absent with “reduced silver colloids” (Figure A17) and also in oPD SERS spectra with gold nanoparticles [37], they should be attributed to some products of oPD oxidation by Ag(I), which exists in some amount in silver colloids as a result of Ag oxidation by atmospheric oxygen. The presence of these bands in the oPD spectrum with silver colloid has previously been observed [15,37]. It should be noted that Koh et al. [37] specifically added 1.6 mM of ascorbic acid to silver colloid as an antioxidant, but it acts as a reducing agent only in alkaline conditions. If ascorbic acid was added without a pH adjustment (as we did for hydroxylamine), it would not prevent nanoparticle oxidation.

The relative intensity of these bands (compared to set A considered above) increases at lower oPD concentrations. At a fixed amount of oxidized silver, fixed amounts of oPD oxidation products will form. At high oPD concentrations (10 mM), there is still plenty of oPD left to successfully compete with oPD oxidation products for binding with the silver surface, resulting in their weak spectrum. At a lower oPD concentration (0.1 mM), a noticeable fraction of oPD is oxidized; thus, the relative spectrum of oxidized oPD becomes stronger.

It is known that both ions of silver (I) [66,67] and silver nanoparticles [68,69] facilitate the oxidation of oPD to DAP. However, the band positions of set B have very little in common with the SERS spectrum of neutral or protonated DAP (Figure A8). We suppose that set B might represent some previously reported intermediate(s) on a route from oPD to DAP [30,54].

Band set C appears in acidic conditions and at lower oPD concentrations. These bands fully correspond to neutral DAP (shadowed blue), DAPH^+^ (purple), or DAPH_2_^2+^ (green). As DAP bands present both with normal and reduced silver colloids, this DAP is not a result of oPD oxidation by silver (I), but instead, is a minor impurity in the stock oPD and the product of spontaneous oPD oxidation upon storage in the solution prior to measurement. Indeed, for freshly prepared oPD stock, the intensity of these DAP bands is more or less constant at any given conditions but gradually increases upon storage of the oPD solution (Figure A18).

The appearance of DAP bands at an acidic pH agrees with our previous statement that only a neutral oPD (with both NH_2_ groups unprotonated) has some affinity for silver. In any conditions when the concentration of this neutral oPD is low (either if the total oPD concentration is low, e.g., at 0.1 mM, or in acidic conditions below its pK_a_ of 4.55 due to protonation), tiny concentrations of DAP appear in the SERS spectrum due to its much higher affinity for silver.

Band set D (marked with a dashed edge in Figure A17) represents products(s) of the reaction between DAP and NH_2_OH (note B and C on Figure A19 are almost identical). They appear on “reduced silver colloids” at pH 2 and below, and one band appears at pH 9.

Conclusions on the SERS spectra of oPD with AgNPs:

- “Pure” SERS spectra of oPD with AgNPs may be obtained only in reducing conditions if the oxidation of metal silver to silver (I) with atmospheric oxygen is suppressed. Under these conditions, the SERS spectrum of oPD corresponds well to a normal Raman spectrum of neutral oPD and the SERS spectrum of oPD with gold (Table A3).
- If oxidized silver is allowed to form, multiple additional bands appear in the pH range of 5 to 9. They correspond to some oxidation product of oPD other than DAP. This is most likely some kind of intermediate(s) on a route from oPD to DAP.
- Only the neutral form of oPD has some affinity for silver. Binding occurs via at least one NH_2_ group (resulting in an intense broad band at around 983 cm^−1^). It is likely that both NH_2_ groups are involved in oPD binding to the silver surface.
- In acidic conditions and at low concentrations of oPD, its spectrum contains (or is even replaced with) the bands of DAP in neutral, DAPH^+^, or DAPH_2_^2+^ forms, depending on the pH. This DAP is not a result of oPD oxidation by silver, but instead, is a minor impurity in the stock oPD and the product of spontaneous oPD oxidation upon storage in the solution.

## Appendix F

The proper aggregation of metal nanoparticles is vital for signal development via the formation of “hot spots” if electrostatically stabilized Ag or Au colloids are used for SERS [70,71]. The easiest way to do so is to vary the concentration of the aggregating agent while keeping the aggregation duration constant. For the purposes of the preceding pH screening, the nonoptimal aggregation state of AgNPs was not relevant, as the relative intensity of DAP versus oPD was followed. Hence, any reasonable concentration of aggregating agent that causes visible particle aggregation was suitable. By contrast, at the current stage of choosing the best pH out of several candidates, the optimal aggregation of AgNPs becomes vital. The second factor, which has already been mentioned, is that at this stage, the detection system is already sensitive enough to measure minor quantities of DAP in stock oPD, so during the optimization of aggregating agent concentration, a blank (the sample without added DAP) should be measured right before or after the sample with DAP, and its signal should be subtracted. The last factor is the selection of the DAP concentration to use during the procedure. This concentration should be low enough not to cause any aggregation on its own without an added aggregating agent. On the other hand, it should be high enough to result in a reliably measured SERS signal. During our preliminary experiments, 100 nM of DAP was chosen to fulfill both of these requirements.

Two series of solutions containing the desired concentration of the aggregating agent, 1 mM of oPD with or without 100 nM DAP, were prepared. Each solution was mixed at a ratio of 1:1 with AgNPs in 5 mM NaCl, and exactly 2 min later, the SERS spectrum was acquired. The resulting curves for the two most intense bands for each pH are presented in Figure A20, Figure A21, Figure A22 and Figure A23.

First, it should be noted that all the data have a high variability. This is caused by the low concentration of DAP used and also by the fact that each data point represents the difference between two measurements (with DAP and without), which additionally increases the uncertainty. Nevertheless, due to the high total number of points, these curves might be used to choose at least some near-optimal conditions.

With H_2_SO_4_ as an aggregating agent (Figure A20), the position of the band around 730 cm^−1^ monotonously shifts from 733 to 730 cm^−1^ with an increasing acid concentration due to the transition from DAPH^+^ to DAPH_2_^2+^. Its intensity rises with added H_2_SO_4_, and we stopped at 1.4 M, as the mixture became too corrosive. The curve for the band 1374 cm^−1^ has a broad maximum from 0.28 to 0.7 M; 0.42 M has been chosen for further work.

With pH 2 glycine buffer (Figure A21), the curve for the band 733 cm^−1^ is essentially constant between 0.2 and 0.7 M, and 0.47 M has been chosen. The curve for the band 1374 cm^−1^, on the other hand, has a pronounced maximum at 0.27 M.

The curves for both bands with a pH 3 citrate buffer (Figure A22) have a broad maximum around 0.93 M. The same is true for pH 4 citrate buffer (Figure A23), with the optimum for both bands being around 1.12 M.

## Appendix G

This appendix demonstrates a very important issue related to quantitative SERS using AgNPs: the loss of nanoparticles due to their adsorption onto laboratory plastic. The production of polypropylene (PP) test tubes, PP tips, and polyethylene (PE) caps via injection molding inevitably requires the addition of some amounts of a so-called “slip agent”, “slip additive”, or “mold release additive” into the plastic. Generally, the substances used as additives could be divided into low-molecular-weight (LMW) (e.g., erucamide and oleamide, fatty acids, LMW esters, and hydrocarbon waxes) and polymeric ones. LMW additives partially migrate to the surface of the product during injection molding. Different test tube/tip manufacturers use different additives with no disclosure of their nature or amount added. As a result, two identical-looking tubes from different manufacturers, both claimed to be made of PP, might hugely differ in their surface properties. Some tubes or tips, in fact, have patches of thin hydrophobic film on their surface. Metallic nanoparticles are known to concentrate on such interfaces [72,73]. This process leads to the loss of AgNPs, especially at elevated ionic strengths, which weaken nanoparticle repulsion. Figure A24 demonstrates some examples of this phenomenon. Under stirring, this process may be very fast (Figure A24c,d), with a rate of 0.5% per minute. Thus far, solutions to this problem are limited. First, we carefully check every type (sometimes even particular lots/batches) of plasticware used for AgNP manipulation. Second, in some cases, 10–15 min of sonication of new tubes in 10 mM NaOH with subsequent rinsing with water has shown good results.

## Appendix H

This appendix contains additional information and illustrations about the quantitative processing of the SERS spectra. A simpler procedure was applied to the spectra obtained under varying conditions (aggregating agent concentration optimization, pH selection, and surface chloride optimization). Its step-by-step application to the spectra is shown in Figure A25 using a dataset from the first repeat of 0 or 500 nM DAP at different pHs (Figure 5b,d).

The second procedure of fitting the spectra with a non-negative linear superposition of known peak shapes was applied to the data obtained in optimized conditions (DAP and HRP calibration curves). Figure A26 illustrates this procedure.

**Table A4:** Equations of peak shapes used for fitting procedures. The x variable is used for Raman shift in cm^−1^.

Band (Figure A26a,b)	Equation
I	$0.9784\times {\left(1+{\left(\frac{x-733.71}{7.694}\right)}^{2}\right)}^{-1.651}\times Exp\left(-0.3799\;ArcTan\left(\frac{x-733.71}{7.694}\right)\right)$
II	$0.9782\times {\left(1+{\left(\frac{x-772.83}{7.15}\right)}^{2}\right)}^{-1.463}\times Exp\left(-0.3595\;ArcTan\left(\frac{x-772.83}{7.15}\right)\right)$
III	$Exp\left(-\frac{1}{2}\times {\left(\frac{x-751.1}{6.98}\right)}^{2}\right)$
IV	$Exp\left(-\frac{1}{2}\times {\left(\frac{x-1333.0}{3.96}\right)}^{2}\right)$
V	$0.395\times {\left(1+{\left(\frac{x-1356.24}{17.33}\right)}^{2}\right)}^{-3.547}\times Exp\left(-3.707\;ArcTan\left(\frac{x-1356.24}{17.33}\right)\right)$
VI	$0.925\times {\left(1+{\left(\frac{x-1374.73}{8.97}\right)}^{2}\right)}^{-1.521}\times Exp\left(-0.6918\;ArcTan\left(\frac{x-1374.73}{8.97}\right)\right)$
VII	$0.991\times {\left(1+{\left(\frac{x-1418.43}{5.51}\right)}^{2}\right)}^{-0.233}\times Exp\left(-0.0938\;ArcTan\left(\frac{x-1418.43}{5.51}\right)\right)$

Peak VII turned out to be very broad, but it is compensated by the polynomial baseline, so the total background for the desired 1374 cm^−1^ band is adequate. At high and moderate concentrations, both procedures result in very close intensities, while in a low concentration range, the second procedure exhibits much less variance for repeated data points. As a result, it is preferred for the developed analytical protocol.

## References

[1] Mekler V.M., Bystryak S.M. (1992). Application of O-Phenylenediamine as a Fluorogenic Substrate in Peroxidase-Mediated Enzyme-Linked Immunosorbent Assay. Anal. Chim. Acta.

[2] Acharya A.P., Nafisi P.M., Gardner A., MacKay J.L., Kundu K., Kumar S., Murthy N. (2013). A Fluorescent Peroxidase Probe Increases the Sensitivity of Commercial ELISAs by Two Orders of Magnitude. Chem. Commun..

[3] Zhang Z., Lai J., Wu K., Huang X., Guo S., Zhang L., Liu J. (2018). Peroxidase-Catalyzed Chemiluminescence System and Its Application in Immunoassay. Talanta.

[4] Liang J., Liu H., Huang C., Yao C., Fu Q., Li X., Cao D., Luo Z., Tang Y. (2015). Aggregated Silver Nanoparticles Based Surface-Enhanced Raman Scattering Enzyme-Linked Immunosorbent Assay for Ultrasensitive Detection of Protein Biomarkers and Small Molecules. Anal. Chem..

[5] Pham X.-H., Hahm E., Kim T.H., Kim H.-M., Lee S.H., Lee S.C., Kang H., Lee H.-Y., Jeong D.H., Choi H.S. (2020). Enzyme-Amplified SERS Immunoassay with Ag-Au Bimetallic SERS Hot Spots. Nano Res..

[6] Lu H., Yang Y., Chen R., Huang W., Cheng H., Liu X., Kong H., Li L., Feng J. (2022). Quantitative Evaluation of Human Carboxylesterase 1 by SERS-ELISA Using a Synergistic Enhancement Strategy Based on Gold Nanoparticles and Metal–Organic Framework. Microchem. J..

[7] Campbell F.M., Ingram A., Monaghan P., Cooper J., Sattar N., Eckersall P.D., Graham D. (2008). SERRS Immunoassay for Quantitative Human CRP Analysis. Analyst.

[8] Chen Y., Cheng H., Tram K., Zhang S., Zhao Y., Han L., Chen Z., Huan S. (2013). A Paper-Based Surface-Enhanced Resonance Raman Spectroscopic (SERRS) Immunoassay Using Magnetic Separation and Enzyme-Catalyzed Reaction. Analyst.

[9] Bozkurt A.G., Buyukgoz G.G., Soforoglu M., Tamer U., Suludere Z., Boyaci I.H. (2018). Alkaline Phosphatase Labeled SERS Active Sandwich Immunoassay for Detection of *Escherichia coli*. Spectrochim. Acta Part A Mol. Biomol. Spectrosc..

[10] Li N., Chen H., Zhang M., Zha Y., Mu Z., Ma Y., Chen P. (2020). A Universal Ultrasensitive Platform for Enzyme-Linked Immunoassay Based on Responsive Surface-Enhanced Raman Scattering. Sens. Actuators B Chem..

[11] Guo W., Hu Y., Wei H. (2017). Enzymatically Activated Reduction-Caged SERS Reporters for Versatile Bioassays. Analyst.

[12] Hu Y., Liao J., Wang D., Li G. (2014). Fabrication of Gold Nanoparticle-Embedded Metal–Organic Framework for Highly Sensitive Surface-Enhanced Raman Scattering Detection. Anal. Chem..

[13] Fu C., Jin S., Shi W., Oh J., Cao H., Jung Y.M. (2018). Catalyzed Deposition of Signal Reporter for Highly Sensitive Surface-Enhanced Raman Spectroscopy Immunoassay Based on Tyramine Signal Amplification Strategy. Anal. Chem..

[14] Dou X., Takama T., Yamaguchi Y., Yamamoto H., Ozaki Y. (1997). Enzyme Immunoassay Utilizing Surface-Enhanced Raman Scattering of the Enzyme Reaction Product. Anal. Chem..

[15] Fu C., Zhang L., Bao M., Zhang Y., Li Y., Wu Y., Jung Y.M. (2022). Signal Amplification Surface-Enhanced Raman Scattering Immunosorbent Assay of Human Chorionic Gonadotrophin Based on Repeated Enzyme Biocatalytic Precipitation. Analyst.

[16] Stevenson R., Ingram A., Leung H., McMillan D.C., Graham D. (2009). Quantitative SERRS Immunoassay for the Detection of Human PSA. Analyst.

[17] Yu Z., Chen L., Wang Y., Wang X., Song W., Ruan W., Zhao B., Cong Q. (2014). A SERS-Active Enzymatic Product Used for the Quantification of Disease-Related Molecules. J. Raman Spectrosc..

[18] Zhan L., Zhen S.J., Wan X.Y., Gao P.F., Huang C.Z. (2016). A Sensitive Surface-Enhanced Raman Scattering Enzyme-Catalyzed Immunoassay of Respiratory Syncytial Virus. Talanta.

[19] Kudryashova A.M., Galstian A.G., Faizuloev E.B., Olenin A.Y., Lisichkin G.V., Zverev V.V., Borisova O.V. (2018). Detection of adenovirus antigen by a surface-enhanced Raman scattering enzyme-linked immunosorbent assay. J. Microbiol. Epidemiol. Immunobiol..

[20] Feng J., Lu H., Yang Y., Huang W., Cheng H., Kong H., Li L. (2021). SERS-ELISA Determination of Human Carboxylesterase 1 Using Metal-Organic Framework Doped with Gold Nanoparticles as SERS Substrate. Microchim. Acta.

[21] Larmour I.A., Faulds K., Graham D. (2010). The Past, Present and Future of Enzyme Measurements Using Surface Enhanced Raman Spectroscopy. Chem. Sci..

[22] Kurochkin I.N., Vasilyeva A.D., Evtushenko E.G., Eremenko A.V., Pergushov D.V., Sigolaeva L.V. (2023). Enzymes in the Development of Physico-Chemical Methods for Biomedical Research. Moscow Univ. Chem. Bull..

[23] Wu Z.-S., Zhou G.-Z., Jiang J.-H., Shen G.-L., Yu R.-Q. (2006). Gold Colloid-Bienzyme Conjugates for Glucose Detection Utilizing Surface-Enhanced Raman Scattering. Talanta.

[24] Hawi S.R., Rochanakij S., Adar F., Campbell W.B., Nithipatikom K. (1998). Detection of Membrane-Bound Enzymes in Cells Using Immunoassay and Raman Microspectroscopy. Anal. Biochem..

[25] Nithipatikom K., McCoy M.J., Kajdacsy-Balla A., Kaul S., Lindholm P.F., Hawi S.R., Rochanakij S., Adar F., Campbell W.B. (1999). Detection of Prostaglandin H Synthase (Cyclooxygenase) -1 and -2 Expression in Single Cancer Cells by Confocal Raman Microspectroscopy. Biomedical Applications of Raman Spectroscopy.

[26] Nithipatikom K., Borscheid C.L., Kajdacsy-Balla A., Kaul S., Lindholm P.F., Pytynia K.B., Campbell W.B., Honn K.V., Marnett L.J., Nigam S., Dennis E., Serhan C. (2002). Determination of Cyclooxygenase and Arachidonic Acid Metabolites in Invasive Human Prostate Cancer Cells. Eicosanoids and Other Bioactive Lipids in Cancer, Inflammation, and Radiation Injury.

[27] Fu C., Wang Y., Tian X., Wu Y., Cao H., Li Y., Jung Y.M. (2021). Horseradish Peroxidase-Repeat Assay Based on Tyramine Signal Amplification for Highly Sensitive H_2_O_2_ Detection by Surface-Enhanced Raman Scattering. Analyst.

[28] Song Y., Wang D., Li Z., Wang L., Fan C., He X., Xu T., Zhang X. (2022). Jigsaw-like Mini-Pillar Platform for Multi-Mode Biosensing. Chin. Chem. Lett..

[29] Tarcha P.J., Chu V.P., Whittern D. (1987). 2,3-Diaminophenazine Is the Product from the Horseradish Peroxidase-Catalyzed Oxidation of o-Phenylenediamine. Anal. Biochem..

[30] Hempen C., van Leeuwen S.M., Luftmann H., Karst U. (2005). Liquid Chromatographic/Mass Spectrometric Investigation on the Reaction Products in the Peroxidase-Catalyzed Oxidation of o-Phenylenediamine by Hydrogen Peroxide. Anal. Bioanal. Chem..

[31] Evtushenko E.G., Gavrilina E.S., Gusarova D.Y., Vasil’eva A.D., Yurina L.V., Kurochkin I.N. (2023). Application of Hydroxylamine Sols of Silver Nanoparticles to Obtain Reference SERS Spectra. Bull. Lebedev Phys. Inst..

[32] Qi G., Fu C., Chen G., Xu S., Xu W. (2015). Highly Sensitive SERS Sensor for Mercury Ions Based on the Catalytic Reaction of Mercury Ion Decorated Ag Nanoparticles. RSC Adv..

[33] Bovaird J.H., Ngo T.T., Lenhoff H.M. (1982). Optimizing the O-Phenylenediamine Assay for Horseradish Peroxidase: Effects of Phosphate and PH, Substrate and Enzyme Concentrations, and Stopping Reagents. Clin. Chem..

[34] Gallati H., Brodbeck H. (1982). Peroxidase Aus Meerrettich: Kinetische Studien Und Optimierung Der Aktivitätsbestimmung Mit Den Substraten H_2_O_2_ Und o-Phenylendiamin. J. Clin. Chem. Clin. Biochem..

[35] Fornera S., Walde P. (2010). Spectrophotometric Quantification of Horseradish Peroxidase with O-Phenylenediamine. Anal. Biochem..

[36] Leopold N., Lendl B. (2003). A New Method for Fast Preparation of Highly Surface-Enhanced Raman Scattering (SERS) Active Silver Colloids at Room Temperature by Reduction of Silver Nitrate with Hydroxylamine Hydrochloride. J. Phys. Chem. B.

[37] Koh T.Y., Greaves S.J., Griffith W.P. (1994). Vibrational Spectra of 1,2-Diaminobenzene, 4,5-Dimethyl-1,2-Diaminobenzene and Catechol and Their SER Spectra. Spectrochim. Acta Part A Mol. Spectrosc..

[38] Ouyang L., Li D., Zhu L., Yang W., Tang H. (2016). A New Plasmonic Pickering Emulsion Based SERS Sensor for in Situ Reaction Monitoring and Kinetic Study. J. Mater. Chem. C.

[39] He D., Jones A.M., Garg S., Pham A.N., Waite T.D. (2011). Silver Nanoparticle–Reactive Oxygen Species Interactions: Application of a Charging–Discharging Model. J. Phys. Chem. C.

[40] Sigg L., Lindauer U. (2015). Silver Nanoparticle Dissolution in the Presence of Ligands and of Hydrogen Peroxide. Environ. Pollut..

[41] Brown K.C., Corbett J.F., Loveless N.P. (1979). Spectrophotometric Studies on the Protonation of Hydroxy and Aminophenazines in Aqueous Solution. Spectrochim. Acta Part A Mol. Spectrosc..

[42] He X., Zhang L., Chua R., Wong P.K.J., Arramel A., Feng Y.P., Wang S.J., Chi D., Yang M., Huang Y.L. (2019). Selective Self-Assembly of 2,3-Diaminophenazine Molecules on MoSe_2_ Mirror Twin Boundaries. Nat. Commun..

[43] Iseminger P.W., Gregory M., Weakley T.J.R., Caple G., Sykes A.G. (1997). Characterization of 3-Aminophenazin-2-Ol Isolated from the Chemical Oxidation of o-Phenylenediamine. J. Org. Chem..

[44] Lin C.-E., Chen Y.-T. (2000). Migration Behavior and Separation of Benzenediamines, Aminophenols and Benzenediols by Capillary Zone Electrophoresis. J. Chromatogr. A.

[45] Arakawa H., Nakabayashi S., Ohno K., Maeda M. (2012). New Fluorimetric Assay of Horseradish Peroxidase Using Sesamol as Substrate and Its Application to EIA. J. Pharm. Anal..

[46] Marzocchi E., Grilli S., Della Ciana L., Prodi L., Mirasoli M., Roda A. (2008). Chemiluminescent Detection Systems of Horseradish Peroxidase Employing Nucleophilic Acylation Catalysts. Anal. Biochem..

[47] Iwata R., Ito H., Hayashi T., Sekine Y., Koyama N., Yamaki M. (1995). Stable and General-Purpose Chemiluminescent Detection System for Horseradish Peroxidase Employing a Thiazole Compound Enhancer and Some Additives. Anal. Biochem..

[48] Sakharov I.Y., Demiyanova A.S., Gribas A.V., Uskova N.A., Efremov E.E., Vdovenko M.M. (2013). 3-(10′-Phenothiazinyl)Propionic Acid Is a Potent Primary Enhancer of Peroxidase-Induced Chemiluminescence and Its Application in Sensitive ELISA of Methylglyoxal-Modified Low Density Lipoprotein. Talanta.

[49] Orendorff C.J., Gearheart L., Jana N.R., Murphy C.J. (2006). Aspect Ratio Dependence on Surface Enhanced Raman Scattering Using Silver and Gold Nanorod Substrates. Phys. Chem. Chem. Phys..

[50] Zannotti M., Rossi A., Giovannetti R. (2020). SERS Activity of Silver Nanosphere, Triangular Nanoplates, Hexagonal Nanoplates and Quasi-Spherical Nanoparticles: Effect of Shape and Morphology. Coatings.

[51] Doyle R.P., Kruger P.E., Mackie P.R., Nieuwenhuyzen M. (2001). Phenazine-2,3-Diamine. Acta Crystallogr. Sect. C.

[52] Mei L., Tai L.S., Tao F.H., Jie S., Rong L.Q. (2012). A Novel Synthesis of 2,3-Diaminophenazine. Res. Chem. Intermed..

[53] Mahato R.K., Mahanty A.K., Kotakonda M., Prasad S., Bhattacharyya S., Biswas B. (2021). A Hydrated 2,3-Diaminophenazinium Chloride as a Promising Building Block against SARS-CoV-2. Sci. Rep..

[54] Li D.-J., Li X.-W., Xie Y.-X., Cai X.-Q., Zou G.-L. (2005). Identification of Intermediate and Product from Methemoglobin-Catalyzed Oxidation of o-Phenylenediamine in Two-Phase Aqueous—Organic System. Biochem..

[55] Badawi H.M., Förner W., Ali S.A. (2013). A Comparative Study of the Infrared and Raman Spectra of Aniline and O-, m-, p-Phenylenediamine Isomers. Spectrochim. Acta Part A Mol. Biomol. Spectrosc..

[56] Kaya Kınaytürk N., Kalaycı T., Tunalı B. (2021). Experimental and Computational Investigations on the Molecular Structure, Vibrational Spectra, Electronic Properties, and Molecular Electrostatic Potential Analysis of Phenylenediamine Isomers. Spectrosc. Lett..

[57] Martin E.L. (1939). O-Phenylenediamine. Org. Synth..

[58] Zhong X., Loges A., Roddatis V., John T. (2021). Measurement of Crystallographic Orientation of Quartz Crystal Using Raman Spectroscopy: Application to Entrapped Inclusions. Contrib. Mineral. Petrol..

[59] Wu D., Fang Y. (2004). Study of Adsorptive Behavior of a Series of N-Aminobenzoic Acids on Silver Nanoparticles by SERS. Sci. Access.

[60] Yan B., Fang Y., Zhao X., Liang L. (2014). A Comparative Study on the Adsorption Behaviors of PABA in the Silver Nano-Particles. J. Mol. Struct..

[61] Akbali B., Yagmurcukardes M., Peeters F.M., Lin H.-Y., Lin T.-Y., Chen W.-H., Maher S., Chen T.-Y., Huang C.-H. (2021). Determining the Molecular Orientation on the Metal Nanoparticle Surface through Surface-Enhanced Raman Spectroscopy and Density Functional Theory Simulations. J. Phys. Chem. C.

[62] Chong N.S., Donthula K., Davies R.A., Ilsley W.H., Ooi B.G. (2015). Significance of Chemical Enhancement Effects in Surface-Enhanced Raman Scattering (SERS) Signals of Aniline and Aminobiphenyl Isomers. Vib. Spectrosc..

[63] Zhao L.-B., Huang R., Bai M.-X., Wu D.-Y., Tian Z.-Q. (2011). Effect of Aromatic Amine–Metal Interaction on Surface Vibrational Raman Spectroscopy of Adsorbed Molecules Investigated by Density Functional Theory. J. Phys. Chem. C.

[64] Tao S., Yu L.-J., Pang R., Huang Y.-F., Wu D.-Y., Tian Z.-Q. (2013). Binding Interaction and Raman Spectra of P–π Conjugated Molecules Containing CH2/NH2 Groups Adsorbed on Silver Surfaces: A DFT Study of Wagging Modes. J. Phys. Chem. C.

[65] Noto R., Leone M., La Manna G., Brugè F., Fornili S.L. (1998). Ab Initio Calculations and Vibrational Spectroscopy on the Phenylenediamine Isomers. J. Mol. Struct. Theochem..

[66] Li F., Liu J., Hu Y., Deng N., He J. (2018). An Ultrasensitive Label-Free Colorimetric Assay for Glutathione Based on Ag+ Regulated Autocatalytic Oxidation of o-Phenylenediamine. Talanta.

[67] Wang D., Lian F., Yao S., Ge L., Wang Y., Zhao Y., Zhao J., Song X., Zhao C., Xu K. (2021). Detection of Formaldehyde (HCHO) in Solution Based on the Autocatalytic Oxidation Reaction of o-Phenylenediamine (OPD) Induced by Silver Ions (Ag+). J. Iran. Chem. Soc..

[68] Al-Onazi W.A., Abdel-Lateef M.A. (2022). Catalytic Oxidation of O-Phenylenediamine by Silver Nanoparticles for Resonance Rayleigh Scattering Detection of Mercury (II) in Water Samples. Spectrochim. Acta Part A Mol. Biomol. Spectrosc..

[69] Abdel-Lateef M.A. (2022). Utilization of the Peroxidase-like Activity of Silver Nanoparticles Nanozyme on O-Phenylenediamine/H_2_O_2_ System for Fluorescence Detection of Mercury (II) Ions. Sci. Rep..

[70] Kleinman S.L., Frontiera R.R., Henry A.-I., Dieringer J.A., Van Duyne R.P. (2013). Creating, Characterizing, and Controlling Chemistry with SERS Hot Spots. Phys. Chem. Chem. Phys..

[71] Pazos-Perez N., Wagner C.S., Romo-Herrera J.M., Liz-Marzán L.M., García de Abajo F.J., Wittemann A., Fery A., Alvarez-Puebla R.A. (2012). Organized Plasmonic Clusters with High Coordination Number and Extraordinary Enhancement in Surface-Enhanced Raman Scattering (SERS). Angew. Chem. Int. Ed..

[72] Edel J.B., Kornyshev A.A., Urbakh M. (2013). Self-Assembly of Nanoparticle Arrays for Use as Mirrors, Sensors, and Antennas. ACS Nano.

[73] Velleman L., Sikdar D., Turek V.A., Kucernak A.R., Roser S.J., Kornyshev A.A., Edel J.B. (2016). Tuneable 2D Self-Assembly of Plasmonic Nanoparticles at Liquid|liquid Interfaces. Nanoscale.

